# Electrically Tunable Friction through Surface Adsorption Layer Restructuring

**DOI:** 10.1021/acsami.5c20376

**Published:** 2025-12-17

**Authors:** Yun Zhao, Zhaoran Zhu, Jie Zhang, Erik Weiand, Chao Wang, James P. Ewen, Daniele Dini, Hugh A. Spikes, Janet S.S. Wong

**Affiliations:** † Department of Mechanical Engineering, 4615 Imperial College London, London SW7 2AZ, U.K.; ‡ State Key Laboratory of Solid Lubrication, 71122 Lanzhou Institute of Chemical Physics, Chinese Academy of Sciences, Lanzhou 730000, China

**Keywords:** tribotronics, active tribology, electrically tunable, aqueous lubricant, cation and anion exchange, adsorption layer restructuring

## Abstract

Electric-potential-controlled friction, which manipulates the frictional response of lubricants via an applied potential, offers the possibility of on-demand lubrication. Conventional understanding suggests that the applied potential influences the adsorption of surfactant ions on rubbing surfaces, thereby altering friction. This study investigates the effect of applied potential on the tribological behavior of sodium dodecyl sulfate (SDS) aqueous solutions in steel–steel contacts through experiments and molecular simulations. It is shown that SDS, as an anionic surfactant, readily forms hemicylindrical surface micelles due to electrostatic and hydrophobic interactions, achieving high coverage even at low concentrations. Consequently, the adsorbed Na^+^ counterions are more responsive to the applied potential than the SDS anions. Contrary to the common belief, friction in steel–steel contacts is governed by Na^+^ concentration through its role in manipulating the structures of the adsorbed SDS aggregates. A critical Na^+^ concentration—achieved either through concentrated SDS solutions or added sodium salt—is required for friction to increase with increasingly negative potential. This friction increase can be attributed to a transition from hemicylindrical to hemispherical surface micelles. This work underscores the competing roles of electrostatic and hydrophobic interactions in surfactant lubrication, suggesting that an effective electro-responsive additive must balance these interactions to enable potential-driven modulation. These findings provide key insights for the design of smart lubricants with potential-tunable friction properties.

## Graphical abstract

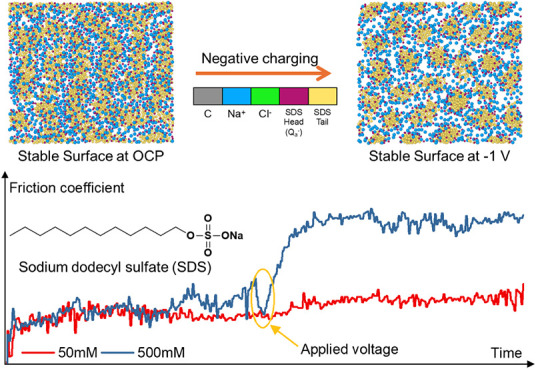

## 1 Introduction

Tribology is a discipline that focuses on studying friction, wear, and lubrication of mechanical systems.^1,2^ Tribology is ubiquitous in our daily lives and prominent in areas such as manufacturing, transportation, and medical technology, to name a few. The ability to effectively manage friction is the key to machine durability and energy efficiency, resulting in substantial economic benefits, estimated to be about 1–2% of the Gross Domestic Product (GDP) on a global scale.^3,4^ Friction of lubricated systems is mainly governed by the properties of lubricants and their interactions with moving surfaces. As a result, lubricant research and development has attracted significant attention, especially regarding additives.^5–9^

Active tribology (also tribotronics)^10^ is a proactive approach that controls friction between dynamic components through external stimuli, thereby enabling manipulation and real-time optimization of the component performance. This strategy has important practical applications. The ability to control friction can enhance the functional range and efficacy of moving components.^11–13^ Promoting ultralow friction is clearly important in a wide range of applications, including automated manufacturing processes and robotic components, where reducing friction and wear enhances efficiency and speed.^14^ On the other hand, applications that require strong grip or slip resistance, such as automotive braking systems, need high friction for rapid and reliable stopping. Precise friction management is crucial in increasing the service life of moving parts.^15^ Consequently, the development of efficient active tribology techniques is crucial in improving the performance of engineering applications and potentially enabling new technology.

In the field of active tribology, a common strategy involves the development of lubricant additives that are responsive to external stimuli.^16–21^ Typical external stimuli include temperature, pressure, velocity, humidity, chemical environment, electrical potentials, magnetic fields, acoustic waves, and light exposure.^22–25^ These external conditions influence the properties of a lubricant, resulting in changes to the lubrication state between two moving surfaces. Among these, electrical potential has received particular interest due to its suitability in real-time monitoring, bidirectional friction regulation, and high-precision manipulation. Furthermore, the ease of applying potentials also enhances their applicability.

The promise of electrical potential-controlled friction has been demonstrated since the pioneering research by Thomas Edison in the late 19th century.^26–36^ Early investigations centered on determining whether the coefficient of friction (COF) could be changed by an applied potential in aqueous salt lubricants. Notable progression occurred in the 1950s with the use of three-electrode systems, enhancing the capability of triboelectrochemistry and triboelectrophysics experimentation. Over subsequent decades, numerous working mechanisms of electrical potential-controlled friction were proposed, including the adsorption of metal cations and chemical species on metal surfaces.^37^ In 1992, Brandon et al. studied the effect of applied potential on the organic additive of neutralized octanoic acid.^26^ They suggested that a positive potential promoted the adsorption of octanoate species, leading to a reduced friction. In the following years, sodium dodecyl sulfate (SDS) and ionic liquids have been used as aqueous and nonaqueous additives where the effect of applied potential on friction performance has been observed.^27–32^

It is widely accepted that potential-controlled friction is primarily attributed to the promotion or inhibition of additive adsorption on metal surfaces.^38^ When a surface potential is applied, a concentration gradient of anions and cations known as an electric double layer is formed in the proximity of the surfaces. For a positive surface potential, the near surface region becomes enriched with anions and depleted of cations, whereas applying a negative potential induces the opposite effect. This impacts friction as this is mainly determined by the structure of the additive layer closest to the metal surface. Theoretical simulations have also demonstrated that changing ionic composition on a metal surface impacts its frictional properties.^39,40^ Previous studies have so far mainly utilized either low additive concentrations or fixed additive concentrations. The impact of the metal surface potential on cation or anion distributions and their effects on the additive adsorption layer and friction remain under-explored. Additionally, most studies used a rubbing pair (such as a ball and a disc) as positive and negative electrodes. The resulting surface asymmetry makes it challenging to confirm which interface affects the frictional properties.

This study combines experimental and computational work to examine the effect of an applied surface potential on the frictional performance of a steel–steel contact in an aqueous SDS solution. A three-electrode system is incorporated in a tribometer such that both surfaces have the same surface potentials, creating a symmetric system. We investigate how SDS concentration influences factors such as sodium ion concentration, conductivity, viscosity, degree of aggregation, chemical state, and pH of a SDS solution and how these affect the sensitivity of SDS tribological performance to the applied potential. Simulations are used to examine the role of the applied potential and cation concentration in inducing morphological changes in the SDS-adsorbed surface film. Together, these approaches offer insights into the origin of electric-potential-controlled friction and guide the development of more effective electrically responsive lubricant additives.

## 2 Materials and Methods

The effect of an applied surface potential on the friction of a steel–steel contact in aqueous SDS solutions is investigated by using a three-electrode system over a wide concentration range. By correlating the tribological performance with the bulk properties of the solutions, we identify the key factors driving the potential-dependent friction response.

### 2.1 Model Lubricants

Sodium dodecyl sulfate (SDS, >99.0%), sodium dodecylbenzenesulfonate (SBS, technical grade), poly (sodium 4-styrenesulfonate) (PSSS, average Mw ∼70,000), octanesulfonic acid sodium salt (>98%), sodium pentanesulfonate (>95%), and sodium chloride (NaCl, ACS reagent) were purchased from Sigma-Aldrich and used as received. For their chemical structural information, see Figure 1b-d. Model lubricants were prepared by using these chemicals as additives in deionized water (DI water) as the solvent. Specifically, 500 mM SDS solution was prepared by dissolving the weighed SDS in DI water, followed by magnetic stirring until a clear solution was achieved. Lower concentration SDS solutions were obtained by diluting the 500 mM SDS solution accordingly. NaCl was also added to prepare model lubricants with various NaCl concentrations.

**Figure 1:**
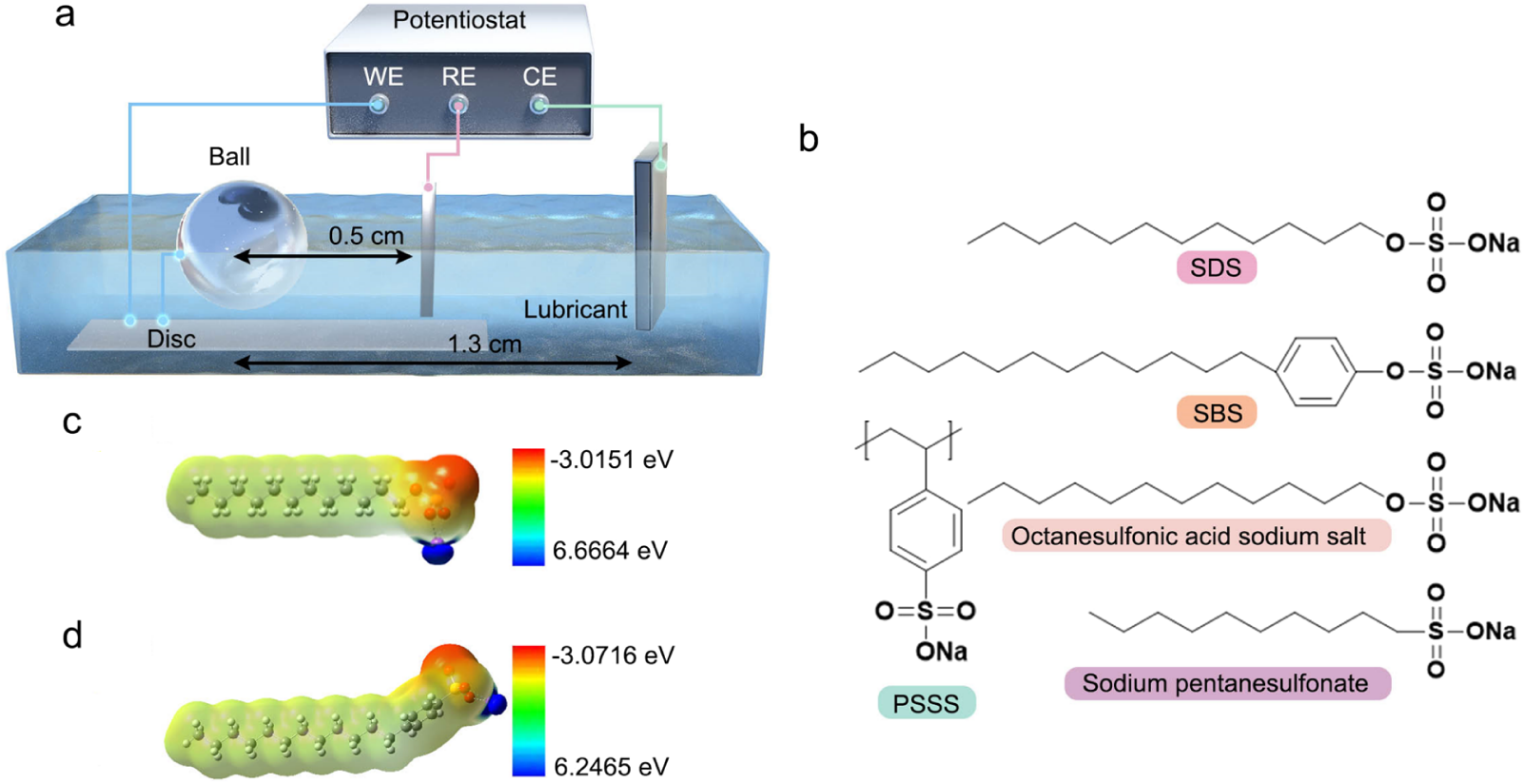
Information on electric potential-controlled friction tests and lubricant additives and their properties. **a.** Schematic diagram of the three-electrode systems with HFRR. **b.** Chemical structures of additives used in this study. **c.** Electronegativity of an SDS molecule. **d.** Electronegativity of a SBS molecule. Abbreviations: WE: Working electrode, RE: Reference electrode, CE: Counter electrode, SDS: Sodium dodecyl sulfate, SBS: Sodium dodecyl benzenesulfonate, PSSS: Poly­(sodium 4-styrenesulfonate).

### 2.2 Tribometer and Test Conditions

For the assessment of frictional performance, a High Frequency Reciprocating Rig (HFRR, PCS Instruments, Acton, UK) was utilized. This instrument creates a rubbing contact by pressing a 6 mm diameter ball in a reciprocating motion against a stationary disc. Through-hardened 52100 steel balls and discs used in our experiments were purchased from PCS Instruments. A three-electrode system in which ball and disc are electrically connected, with their potential controlled via a reference electrode using a Autolab PGSTAT100 potentiostat, was used to regulate electrical potential of the rubbing pair, as illustrated in Figure 1a. Some experiments were conducted with a two-electrode system for validation (Figure S1).

Both the three- and two-electrode systems were configured with the working electrode connected to the rubbing pair and a Pt foil selected as the counter electrode. The distance between the counter electrode and the working electrode was set at 1.3 cm for most tests, but a separation of 0.8 cm was also tested and showed that the electrode distance does not significantly impact the results. With the three-electrode system, a standard electrode functioned as the reference electrode (positioned 0.5 cm from the rubbing pair’s center). The typical HFRR test conditions are shown in Table 1. All rubbing tests were carried out at 25 °C, which is above the Krafft point of SDS (∼15 °C).^41^ The testing parameters produced a Hertzian mean contact pressure *P*_mean_ of approximately 0.55 GPa and a lubricant film thickness of about 2 nm, estimated based on the Hamrock & Dowson Equation.^42^ Thus, our tests operated in a boundary lubrication regime. Some HFRR tests also utilized stainless steel discs and zirconia balls (ZrO_2_, PCS Instrument) under the same test conditions. All specimens were purchased before 2024 and were ultrasonically cleaned in heptane for 10 min, followed by soaking in heptane for overnight. Before use, they were wiped with toluene-soaked tissue and rinsed with toluene.

**Table 1:**
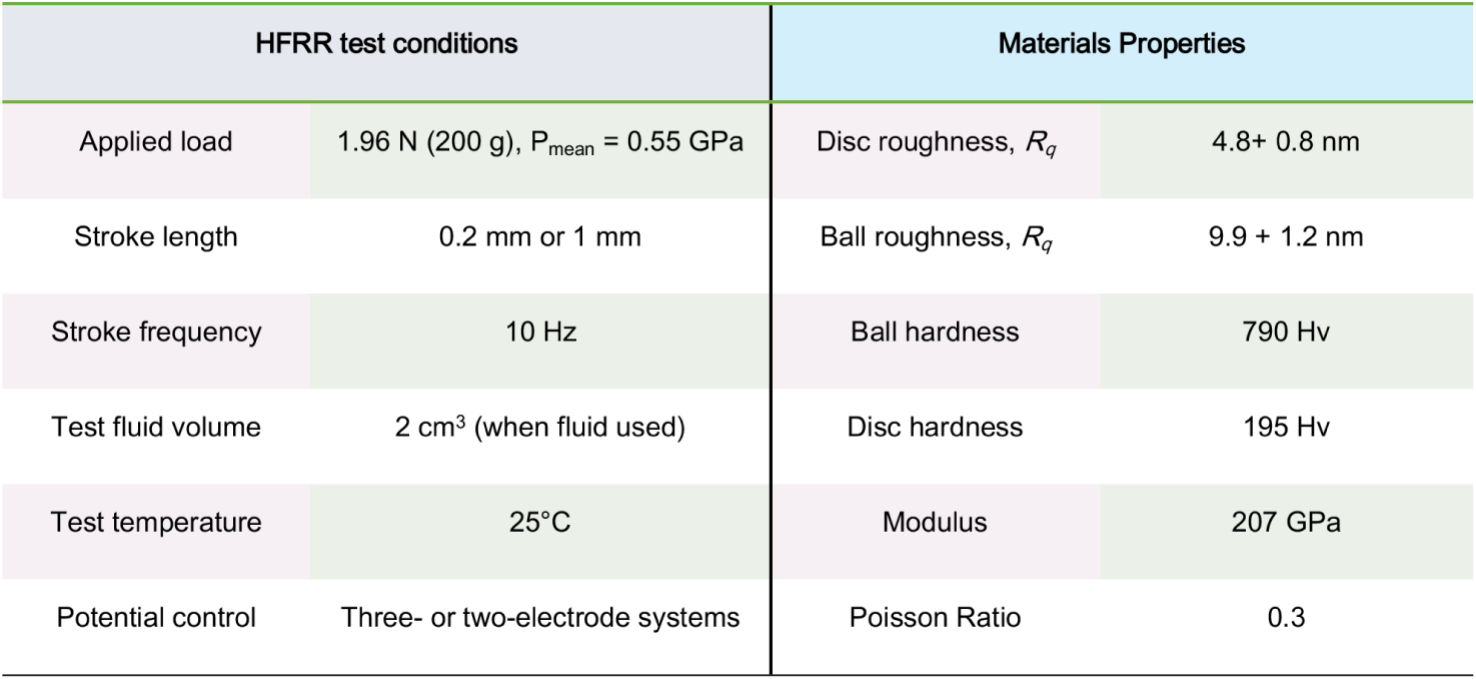
HFRR Test Conditions and Material Properties of Steel Samples

### 2.3 Material and Surface Characterization

After friction tests, wear scars were left on the rubbing surfaces. A RH-2000 digital microscope (Hirox, Tokyo, Japan) was used for the observation of wear tracks on the discs. Prior to imaging, each disk was cleaned with toluene and then wiped with toluene-moistened tissue to remove surface contaminants. Due to the use of reciprocating tests with a ball-on-disc geometry, the sliding speed and the depth of the wear track varied along the track. Hence, instead of wear volume or wear rate, wear track width was used to characterize surface wear, as it is deemed a more reliable measure.

The electrical conductivity of the lubricants was assessed by using a Malvern Zetasizer (Nano ZS, Malvern Instruments Ltd., GB). Viscosity measurements were conducted using a rotating viscometer (NDJ-8S, Nirun, Shanghai, China). The pH values of the solutions were determined with a pH meter (FE28, Mettler Toledo, China). All measurements were repeated three times, and the average value was recorded. A potentiostat (Autolab, PGSTAT100N, Metrohm, Switzerland) measured the open-circuit potential (OCP) in a three-electrode system, while its cyclic voltammetry function was used to assess the electrochemical stability of the lubricants. High-resolution transmission electron microscopy (HRTEM, Tecnai G2 F30, FEI, United States) was used to investigate the size and quantity of aggregates in the lubricant solutions at an accelerating voltage of 300 kV and a resolution of 2 Å. Following the sample preparation protocol of ref 43 for micelle investigation, test samples were prepared by placing drops of lubricant onto carbon-coated copper grids, which were placed on a hydrophilic glass fiber paper and then heated under an infrared lamp for 15 min. A microlaser confocal Raman spectrometer (Horiba LabRAM HR800, France) equipped with a 532 nm laser provided information about the chemical state of additives in different lubricants.

Adsorption of SDS lubricants on metallic surfaces was examined using a quartz crystal microbalance with an electrochemistry module (QCM-E, QSense Explorer, Biolin Scientific),^44^ which operated at 40 °C with a flow rate of 0.1 mL/min and used a gold-coated sensor under the manipulation of electrical potentials via a three-electrode system. Before testing, the QCM holder and tubing were rinsed three times with deionized water and dried in a 60 °C oven. The QCM sensor was first rinsed three times with deionized water, then soaked in ethanol, and ultrasonicated for 30 s. After drying, it was treated with UV-ozone for 5 min. Tests were conducted immediately. Deionized water was first flowed into the QCM module until the signals stabilized. This was followed by flowing the test solution to examine the adsorption behavior of SDS. Finally, deionized water was flowed into the cell to rinse off excess SDS on the sensor surface.

The spatial distribution of elements on metal surfaces exposed to SDS lubricants, both with and without applied potentials, was characterized by using time-of-flight secondary ion mass spectrometry (ToF-SIMS).

### 2.4 Modeling Approach and Computational Methods

Simulations are used to examine the effects of the applied potential and cation concentration on the structure and morphology of adsorbed SDS layers. Results are then correlated with experimental findings to provide insight into the origin of applied potential responsive friction. A brief description of the simulations is provided below. Details can be found in the Supporting Information.

#### 2.4.1 Electronegativity of SDS and SBS

All *ab initio* calculations in this study were conducted using Gaussian 16 software. The geometry optimizations and vibrational frequency calculations were performed employing hybrid density-functional theory (DFT) at the B3LYP/6-311++G­(d, p) level. Grimme’s D3 dispersion correction was applied throughout the computational process. A thermodynamic correction factor of 0.9828 was utilized in these calculations. The solvent environment was modeled using the water solvent model available in the Gaussian 16 package, with solvent parameters detailed in reference.^45^

#### 2.4.2 Computational Method for SDS Behaviors on Rubbing Surface

Coarse-grained molecular dynamics (MD) simulations were used to investigate the effect of electric potentials on the surface behaviors of the anionic surfactant SDS. The constructed systems have approximate dimensions of 20 nm in the *x*- and *y*-directions (Figure 2a). Two graphene sheets, which have been shown to result in similar adsorption behavior to gold and steel, are used as the model surfaces.^46–48^ They are 23 nm apart along the *z*-direction. The model lubricant contains NaCl, SDS, and water. The simulation focuses on the impact of surface potential on the SDS layer adsorbed on the graphene surface (Figure 2b) and its proximity. This is about 3 nm thick in our simulations, consists of three parts: the inner Helmholtz plane (IHP), the outer Helmholtz plane (OHP), and the diffusion layer.

**Figure 2:**
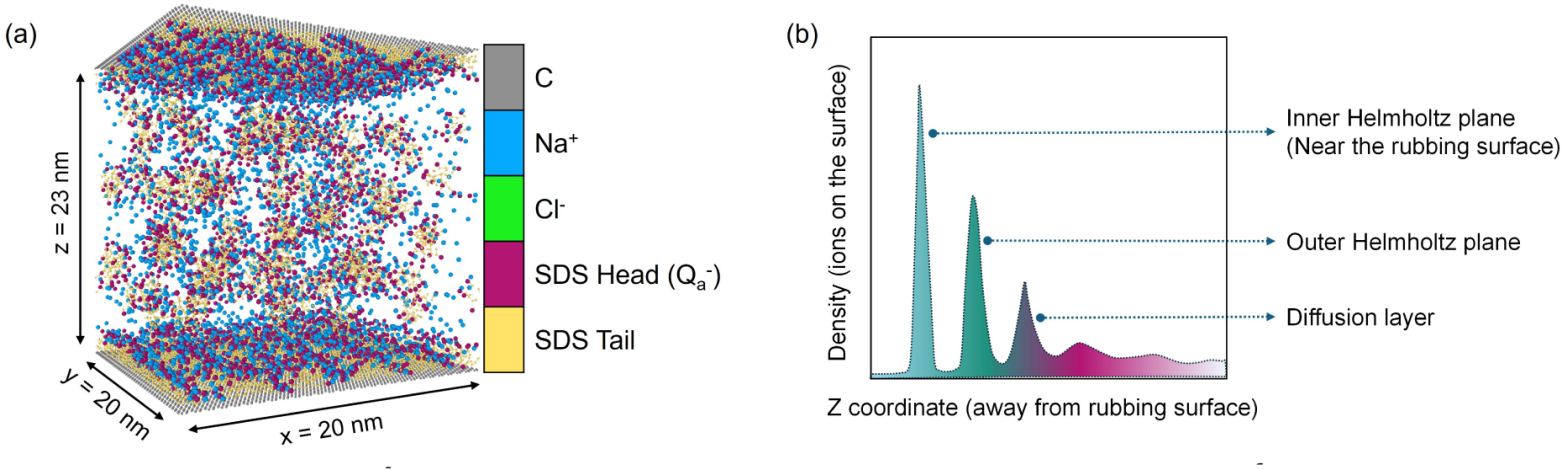
**a.** Schematic of the MD simulation system. **b.** Schematic of the structure of a liquid in the proximity of a charged surface.

The MARTINI 2 force field^49,50^ along with the polarizable MARTINI water model by Yesylevskyy et al.^51^ was employed. This set of MARTINI models was successfully applied to study the adsorption behaviors of various surfactants on a graphene surface.^52,53^ In the MARTINI model, generally, four heavy atoms were represented by a single coarse-grained interaction bead (4:1 mapping). SDS was described by a negatively charged (*Q*_a_) bead head with three apolar C_1_ beads for the hydrocarbon chain and a positively charged sodium counterion (*Q*_d_). For the effect of the addition of salt (NaCl), the negatively charged hydrated chloride anion was presented by the *Q*_a_ bead type. These molecules were confined between two flat graphene sheets composed of MARTINI C_1_ beads. These hydrophobic surfaces are representative of the steel^54^ and gold^55^ surfaces used in the HFRR and QCM experiments, respectively, that quickly become covered by a hydrocarbon layer when exposed to air.^56^ Moreover, hemicylindrical micelles have been observed to form for SDS above the critical micelle concentration (8 mM) on steel,^57^ gold,^58^ and graphite^59^ surfaces, suggesting that aggregation behavior is insensitive to the hydrophobicity of the surfaces. Systems of 150 mM SDS, and 10 mM SDS with 140 mM NaCl were constructed using PACKMOL^60^ and Moltemplate.^61^

All MD simulations were performed in the large atomic/molecular massively parallel simulator (LAMMPS) software.^70^ Velocity Verlet integration using a global Nosé–Hoover^62–64^ scheme was used. A fixed boundary was used in the *z*-direction (thickness direction), and periodic boundary conditions were applied in the *x*- and *y*-directions. The system was first equilibrated at 300 K and 0.1 MPa for 0.5 ns. We ran the uncharged system in the canonical (NVT) ensemble for 200 ns with a time step of 20 fs. After that, the surface-adsorbed SDS structure was stable. Electric potentials were applied to the graphene sheets at the last time step of the uncharged system, using the constant potential method from the ELECTRODE^65^ package in LAMMPS. The electroneutrality of the system was constrained when the graphene sheet was charged. Charged systems were run in the NVT ensemble at 300 K for at least another 200 ns. Further details on the simulation method can be found in the Supporting Information.

## 3 Results and Discussion

SDS is an anionic surfactant known for its exceptional water solubility, with a solubility exceeding 500 mM. The formation of SDS micelles begins at concentrations of around 8 mM. With a further increase in concentration, the aggregation structure of SDS changes from micellar to hexagonal and finally to crystals.^30^ In the concentration range of 50–500 mM, SDS mainly exists in micellar form with nanocrystals. It has been shown that SDS in the range of concentration studied adsorbed in the form of semicylindrical micelles.^30^ Notably, SDS adsorbs on surfaces even at extremely high or low concentrations in water.^29^ To maintain electroneutrality, the cations are in the proximity of the anions in the adsorbed layer. Upon a change of the metal surface potential, the adsorbed film may rearrange. These properties may affect the tribological performance of SDS solutions, as shown below.

### 3.1 Frictional Performance of SDS Solutions

Figure 3 shows that the friction coefficient of steel–steel contacts in SDS solutions is much lower than that in water (about 0.55), indicating that the SDS adsorbed layer is effective in friction reduction. At the open circuit potential (OCP) state, all SDS solutions yield a similar coefficient of friction (COF, μ_OCP_) irrespective of their concentrations (Figure 3a). The impact of the applied negative potential on the COF depends on the SDS concentration. For low SDS concentrations, COF at −0.6 V (μ_‑0.6V_) is similar to μ_OCP_. When the SDS concentration is above 100 mM, μ_–0.6V_ rises with SDS concentration (Figure 3b). On the other hand, the application of a positive potential shows little effect on COF irrespective of SDS concentration (Figure 3c). Note also that the width of wear tracks on rubbed discs increases slightly with SDS concentrations beyond 100 mM (Figure 3d; see also Figure S2). It is likely that an applied potential may change the structure and/or morphology of the SDS adsorbed layer, giving rise to a change in friction. Our results however imply that only changes due to a negative surface potential can influence friction.

**Figure 3:**
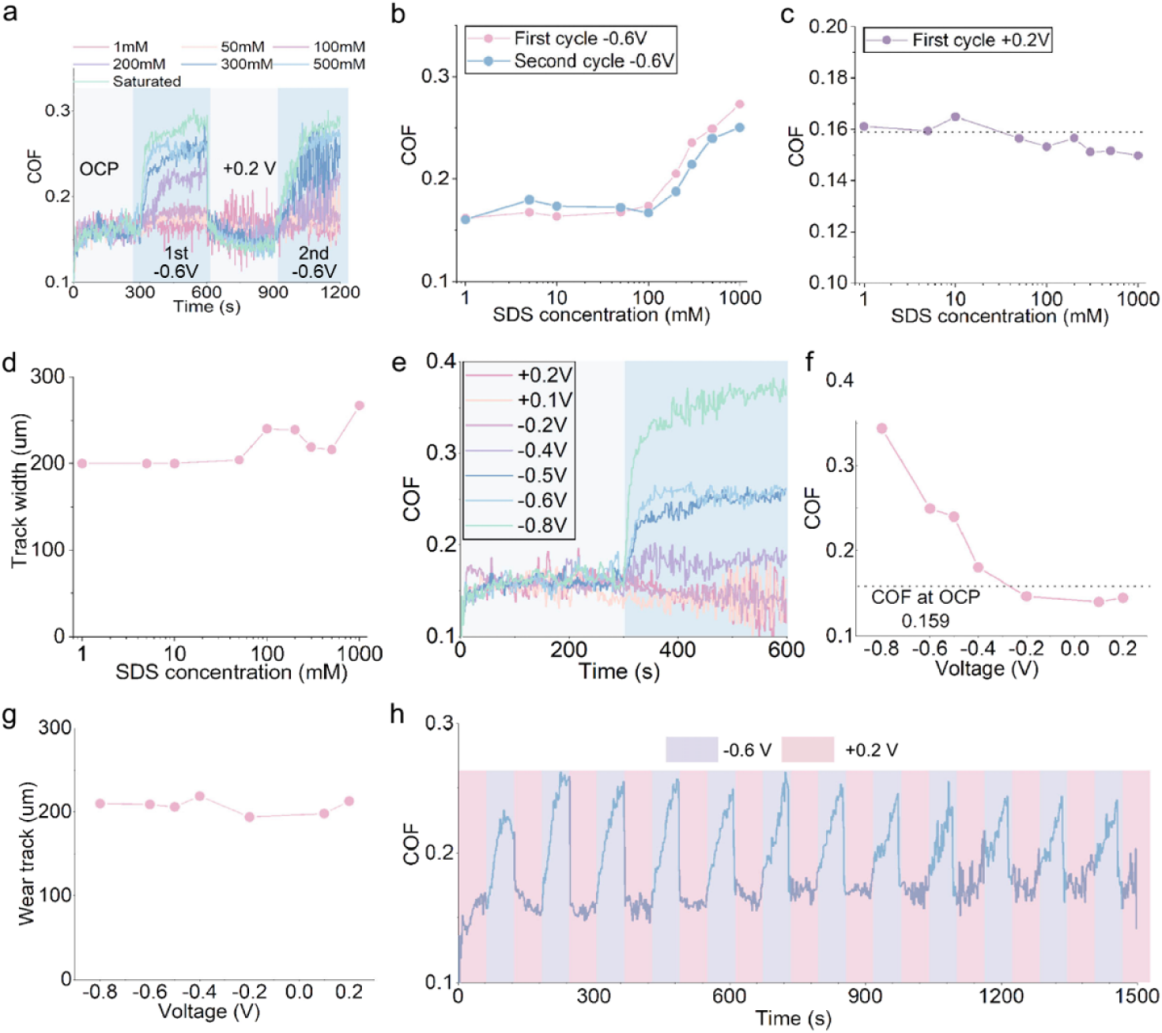
Frictional performance of SDS solutions using a three-electrode system. **a.** The COF of steel–steel contacts in SDS solutions. The potential stated in the figure, for example, “–0.6 V” means −0.6 V was added to the OCP of the reference electrode measured in a 1 mM SDS solution. For example, if an Ag/AgCl reference electrode exhibits a stable OCP of −0.33 V in a 1 mM SDS solution, we set the test potential to −0.93 V. **b.** Average COF of SDS solutions at −0.6 V from (a). **c.** Average COF of SDS solutions at +0.2 V from (a). **d.** Wear track width of discs obtained after tests in (a). **e.** COF of 500 mM SDS solution under various applied potentials. **f.** Average COF of 500 mM SDS solution under various potential conditions from (e). **g.** Wear track width of discs obtained after tests in (e). **h.** Cyclic performance of 500 mM SDS solution at −0.6 V and +0.2 V.

The effects of applied potential on COF are illustrated using a 500 mM SDS solution; see Figure 3e and f. When the potential is between 0.2 and −0.2 V, the COF is quite constant. From −0.2 to −0.8 V, the COF increases gradually, suggesting a critical negative potential exists. The applied potential does not have a strong effect on the amount of wear (Figure 3g). This suggests that at high SDS concentration, while the application of negative potential may alter the morphology and/or properties of the SDS adsorbed layer and affect friction, the altered SDS layer may still offer wear protection. The effect of the applied potential on the COF is mostly reversible (Figure 3h). With the increasing cycle number, the difference in COF between positive and negative potentials decreases, likely due to progressive surface damage. Similar effects were also observed with a two-electrode system (Figure S4a,b, and c).

The effect of a negative surface potential on friction in an SDS solution is not limited to steel–steel contacts. A similar effect is observed with ZrO_2_ ball/standard steel disc and standard steel ball/440c stainless steel disc rubbing pairs using 500 mM SDS solutions (Figure S5 and S6).

### 3.2 Characterizations of SDS Solutions

To find the drivers behind the effects of surface potential and concentration on COF of steel–steel contacts in SDS solutions, properties of SDS solutions are characterized (Figure 4a-h). The cyclic voltammetry curve of a 500 mM SDS solution using a three-electrode system reveals no significant electrochemical reactions within −1.0 V to −0.5 V compared with other potential regions (Figure 4a), i.e., the solution is electrochemically stable within the applied potential range. This suggests that the observed change in COF with applied potential shown in Figure 3 is unlikely due to electrochemical reactions.

**Figure 4:**
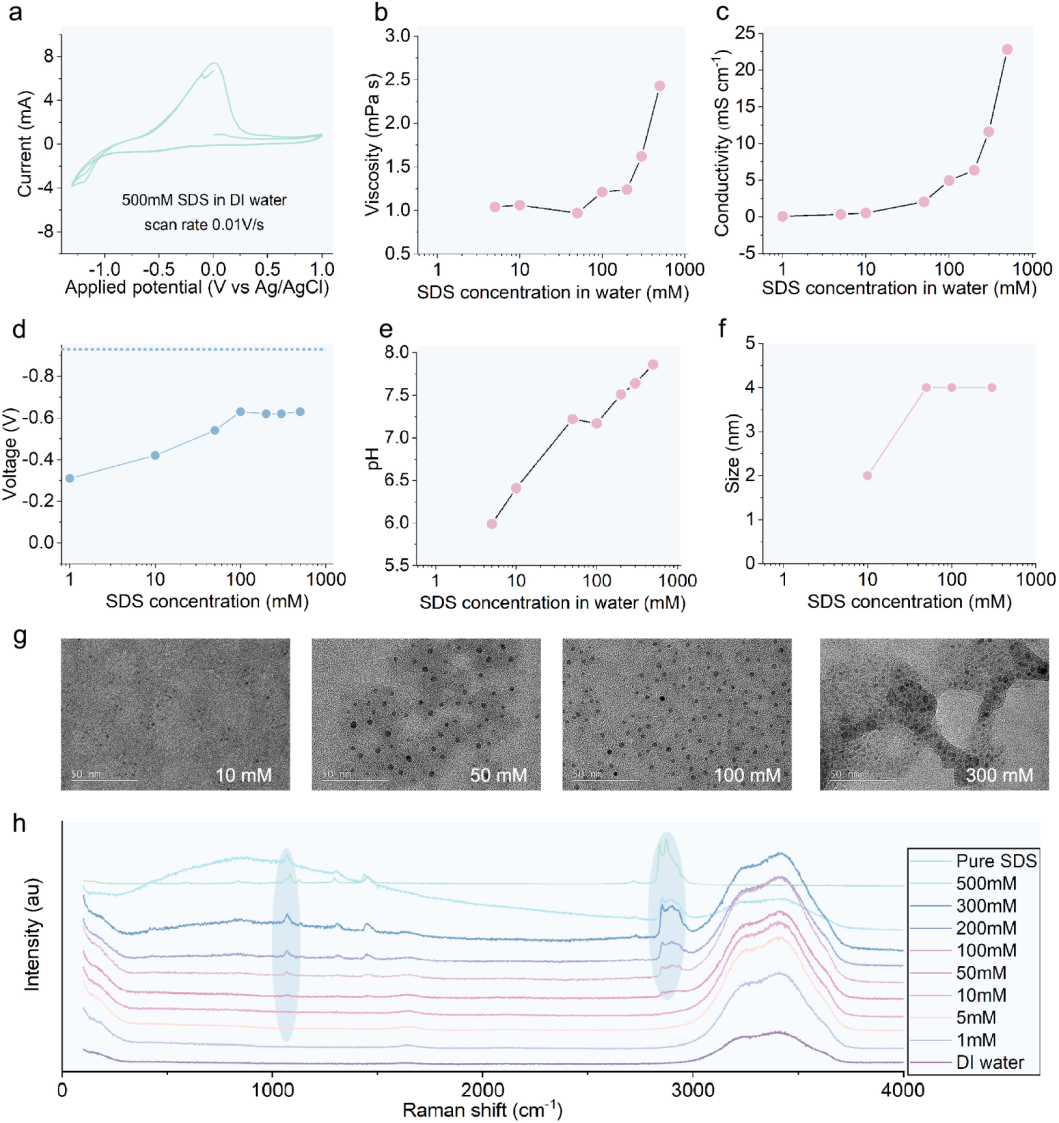
Characterizations of the SDS solutions. **a.** The cyclic voltammetry curves. **b.** Viscosity. **c.** Conductivity. **d.** Open circuit potential (the dotted line represents the set testing potential). **e.** pH. **f.** SDS aggregates size and corresponding TEM images are shown in **g**. **h.** Raman spectra of SDS solutions.

The effect of SDS concentration on bulk properties of SDS solutions shows an interesting trend that pH of the solution increases monotonically as the SDS concentrations increase (Figure 4e). On the other hand, the open-circuit potential increases and then stabilizes when the SDS concentration exceeded 100 mM (Figure 4d). High-resolution transmission electron microscopy reveals that the average size of SDS aggregates rises with SDS concentrations and then plateaus when the SDS concentration is higher than 50 mM, (Figure 4f and g). Raman spectra of SDS solutions at concentrations above 100 mM show peaks at 1100 cm^–1^ and 2900 cm^–1^, which coincide with the peaks of SDS crystals (Figure 4h). Interestingly, its viscosity and conductivity increase sharply with concentration beyond 100 mM (Figure 4b and c), similar to the effect of SDS concentration on COF at an applied negative potential (Figure 3b). These results indicate that the conductivity and/or viscosity of a SDS solution may be responsible for the sensitivity of its COF to an applied negative potential.

### 3.3 Effect of Na^+^ Ion Concentrations on Friction

Since the frictional response of the SDS solution is more sensitive to negative than positive potential, the cations in the solution may play a crucial role in our observations. To test the hypothesis, various amounts of NaCl were added to 10 mM SDS solutions. An applied negative potential of −0.6 V does not affect the COF of a 10 mM SDS solution (Figure 5a). An addition of NaCl to the 10 mM SDS however increases μ_–0.6V_ when NaCl concentrations are 100 mM or higher (Figure 5a and b). Comparing the results in Figure 5b with those in Figure 3b, this shows that the addition of NaCl enables low-concentration SDS solutions to behave like a high-concentration SDS solution of similar Na content.

**Figure 5:**
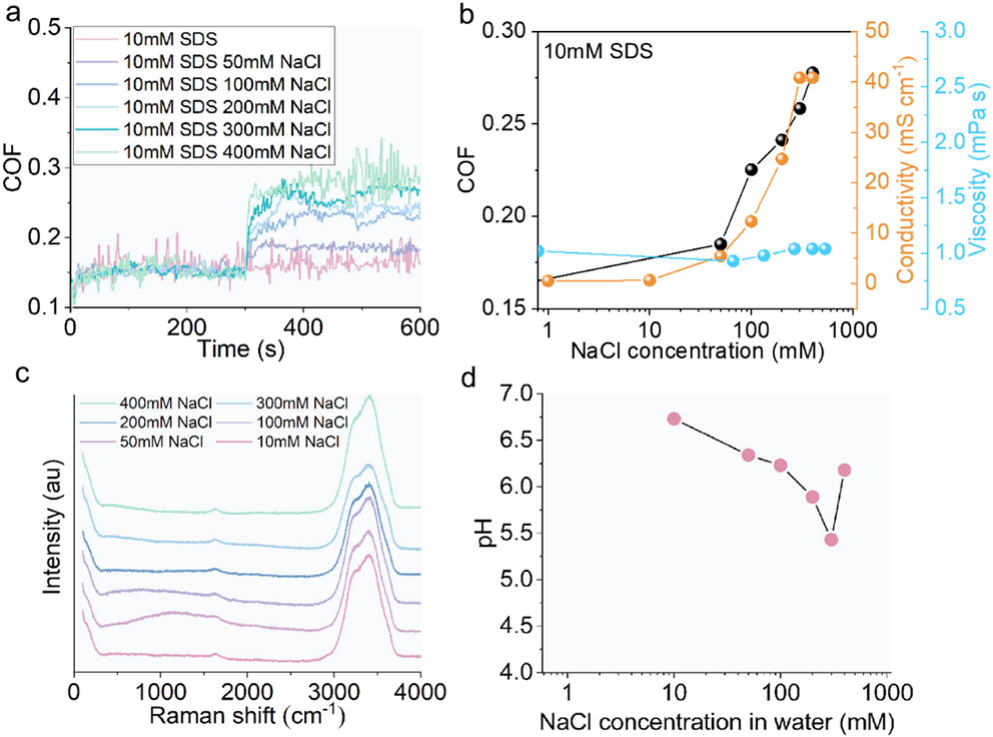
Frictional performance and physical properties of a 10 mM SDS solution with various NaCl concentrations. **a.** COF of 10 mM SDS solution with various NaCl concentrations; −0.6 V was applied at time = 300 s. **b.** How the COF at −0.6 V, conductivity, and viscosity of 10 mM SDS solution change with NaCl concentrations. **c.** Raman spectra of 10 mM SDS solutions with various NaCl concentrations. **d.** pH of 10 mM SDS solutions with various NaCl concentrations.

Since NaCl is used, the role of Cl^–^ ions needs clarification. At the OCP, interactions between Cl^–^ and the SDS adsorbed layer is likely minimal (see Figure 5a). Under a negative potential, Cl^–^ is repelled from the surface. When a positive potential is applied, SDS anions adsorb more strongly and cover the surface, and thus, Cl^–^ is unlikely to significantly influence the SDS adsorbed layer. Indeed, the effect of positive potential on the COF of SDS solutions with NaCl is small (see Figure S3). Overall, the role of Cl^–^ is likely to be insignificant in this study.

The effect of pH requires further discussion. Steel in low pH solutions is susceptible to corrosion. Note that the COFs at −0.6 V of SDS and SDS with NaCl solutions increase with Na^+^ concentration in a similar manner, while how their pH changes with Na^+^ concentration differs. Additionally, the COF of the SDS solution at the OCP is insensitive to its concentration. This suggests that the pH of the solutions, and hence corrosion, does not play a significant role in the COF of SDS solutions.

### 3.4 Frictional Performance of Other Additive Solutions

Apart from SDS, the COFs of PSSS, sodium pentanesulfonate, octanesulfonic acid sodium salt, and SBS solutions also increase under −0.6 V when a critical additive concentration is reached (Figure 6a-d). This shows that an increase in friction with negative applied potential can occur in many additives in aqueous solutions. Results of dilute PSSS and SBS solutions (10 mM and 12.5 mM, respectively), which have conductivity less than 1 mS cm^–1^, indicate that high conductivity is not necessary for the electrically responsive behavior of the solutions (see Figure 6a and d).

**Figure 6:**
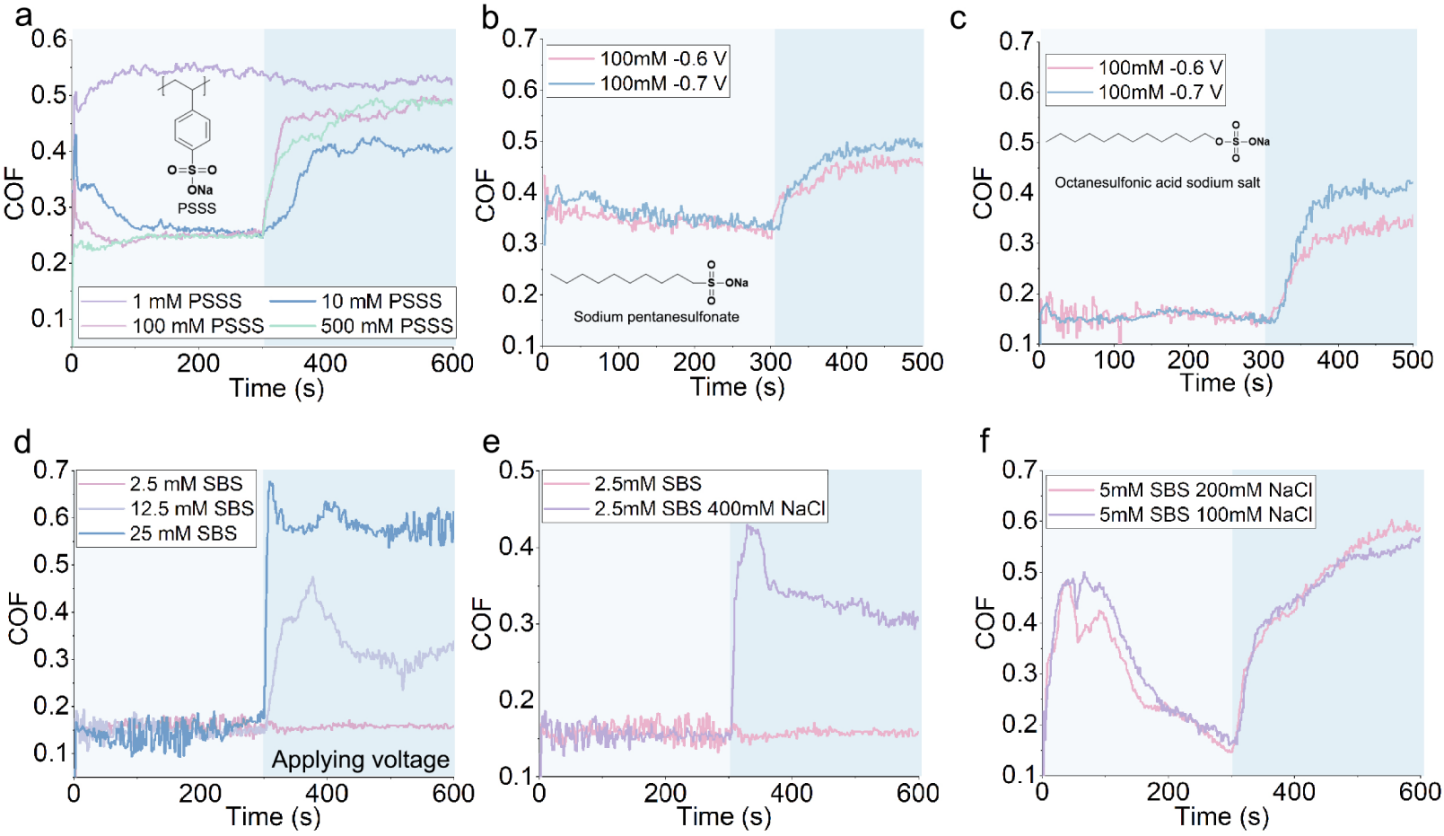
Effect of applied −0.6 V on the COFs of **a**. PSSS, **b**. sodium pentanesulfonate, **c**. octanesulfonic acid sodium salt, and **d.** SBS solutions. **e.** 2.5 mM SBS solution with and without 400 mM NaCl. **f.** COF of 5 mM SBS solution with 100 and 200 mM NaCl. Negative potential was applied in the part of the graphs highlighted in darker blue.

Results from SDS solutions (Figures 3, 4, and 5) suggest that the sensitivity of μ_–0.6V_ of SDS with concentration is likely due to the role of Na^+^ ions. This is confirmed with SBS solutions where higher μ_–0.6V_ is seen at 2.5 mM SBS when 400 mM of NaCl is added (Figure 6e). Recall that μ_–0.6V_ of SBS increases substantially over a modest concentration range (Figure 6d) with minimal change in electrical conductivity (see Figure S7 for bulk properties of SBS). This suggests that the Na^+^ effect on increasing μ_–0.6V_ of SBS and SDS solutions is unlikely to be due simply to a change in the conductivity of solutions.

### 3.5 Reaction Time to an Applied Potential

Previous studies have shown that concentrated NaCl aqueous solutions increased COF under negative potential, while COF is reduced under positive potential conditions.^66^ This was because movements of both Na^+^ or Cl^–^ ions can be manipulated easily by the electric field alone. Thus, the formation of cation- or anion-dominated interfacial layers under electric fields remains a primary factor governing frictional performance. In our test conditions, however, the COF of water and NaCl_(aq)_ in OCP are similar (Figure 7c vs a and b). Unlike previous studies,^66^ the effect of applied potential on COF of NaCl_(aq)_ is weak, irrespective of NaCl concentration (Figure 7a and b). This suggests that the increased sensitivity of the COF of SDS_(aq)_ on the applied potential with the addition of NaCl is not solely due to additional Na^+^, but rather the interaction of these Na^+^ with the SDS adsorbed layer. Such interaction may alter the nature of the SDS adsorbed film. Figure 7c shows that the COF reduces rapidly when water is replaced by an SDS solution as the lubricant. This shows that the formation of SDS layers in OCP condition is rapid. Note that the increase in COF due to an applied negative potential takes time (*t*_–6V_) and depends on the concentration of surfactant (Figure 5a, Figure 6a and d). More concentrated solutions take shorter *t*_–6V_ to achieve a stable μ_–6V_, perhaps due to the higher concentration of Na^+^ ions.

**Figure 7:**
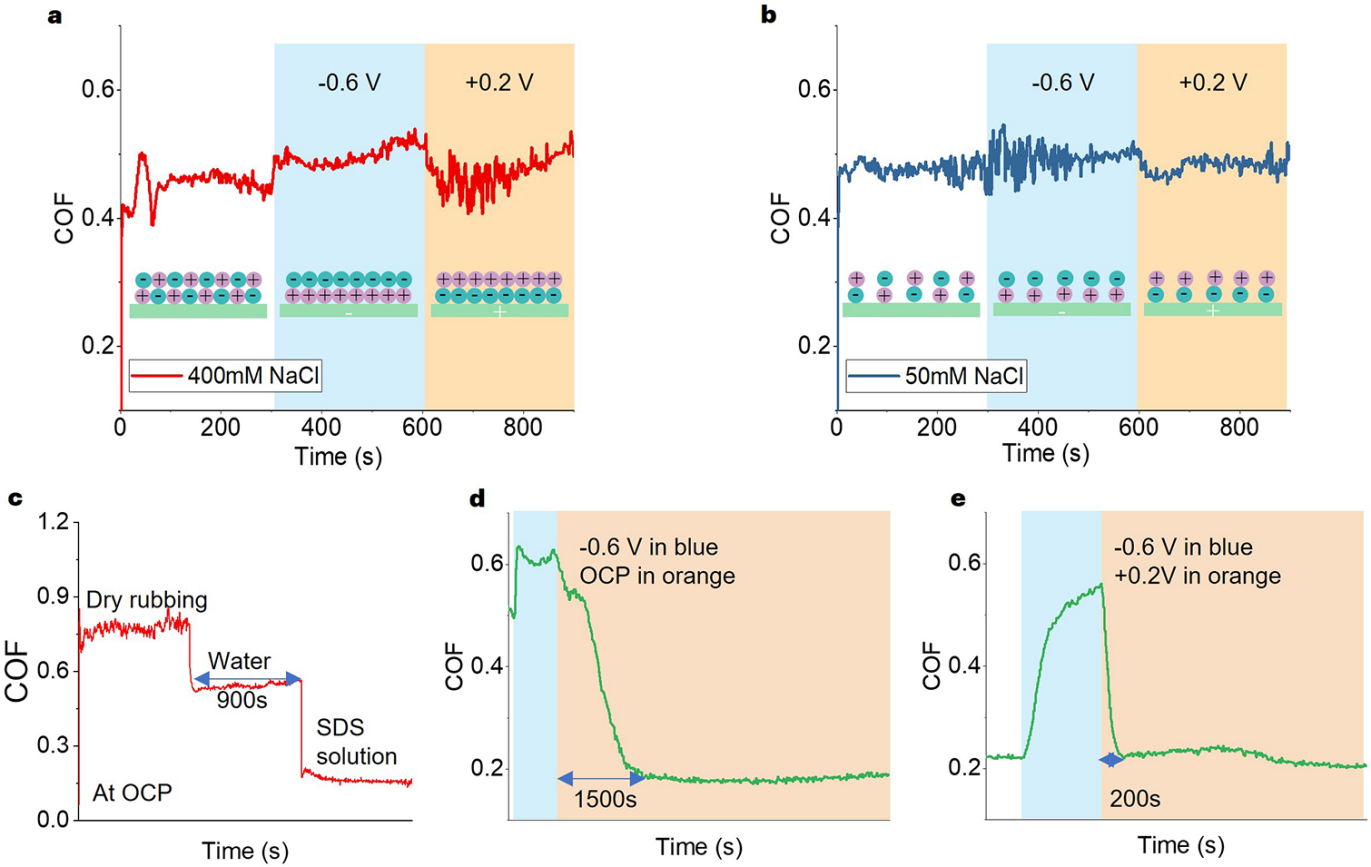
COF of **a.** 400 mM NaCl solution under applied potentials. **b.** 50 mM NaCl solution under applied potentials. **c.** A steel–steel contact at the OCP in dry rubbing, followed by an addition of water, and then by 500 mM SDS solution. **d.** 25 mM SBS solution where the surface potential switches from −0.6 V to the OCP. **e.** 25 mM SBS solution potential when applied potential switches from −0.6 V to +0.2 V.

If the applied potential does not change the adsorbed SDS layer permanently, the nature of adsorbed surfactant layers, and hence the COF, may revert to its original state when the applied potential is removed. This is illustrated using adsorbed SBS layers, which show that the time for the reversion process (*t*_R_ = 1500 s) at OCP is quite long (Figure 7d). *t*_R_ can be substantially shortened if a positive potential is applied (*t*_R_ = 200 s, Figure 7e). This suggests that the restoration of the low friction SBS layer involves the removal of sodium ions in the vicinity of the SBS layer and confirms the role of Na^+^ in manipulating the surfactant adsorbed layer and hence friction.

### 3.6 Adsorption Film Characterizations

Quartz Crystal Microbalance (QCM-D) data in Figure 8a present how frequency (*f*) and dissipation (*D*), for overtone *n* = 3 to 13, of a gold-coated quartz crystal sensor change as the concentration of SDS solution increases in OCP conditions. There is a significant increase in SDS adsorbed mass with concentration, reaching up about 20,000 ng cm^–2^ with 500 mM SDS_(aq)_ (Figure S8). Note that there is a spread of *f* and *D* from different overtones. This differs from conventional surfactant self-assembled monolayers. The spread widens with increasing concentration, which suggests that SDS layers are not rigid and may consist of aggregates at high concentrations on the metal surface.^67^ This is consistent with research that shows that SDS forms micelles on metal surfaces when its concentration is above 8 mM.^30^ Applying a negative potential using the QCM-Electrochemistry (QCM-E) module has a minimum impact on the mass and properties of the SDS adsorbed layer (Figure 8b). This suggests that the increased COF observed with a negative potential in SDS solutions (Figure 3f) is not due to reduced SDS adsorbed mass but rather a change in composition or structures may have been at play.

**Figure 8:**
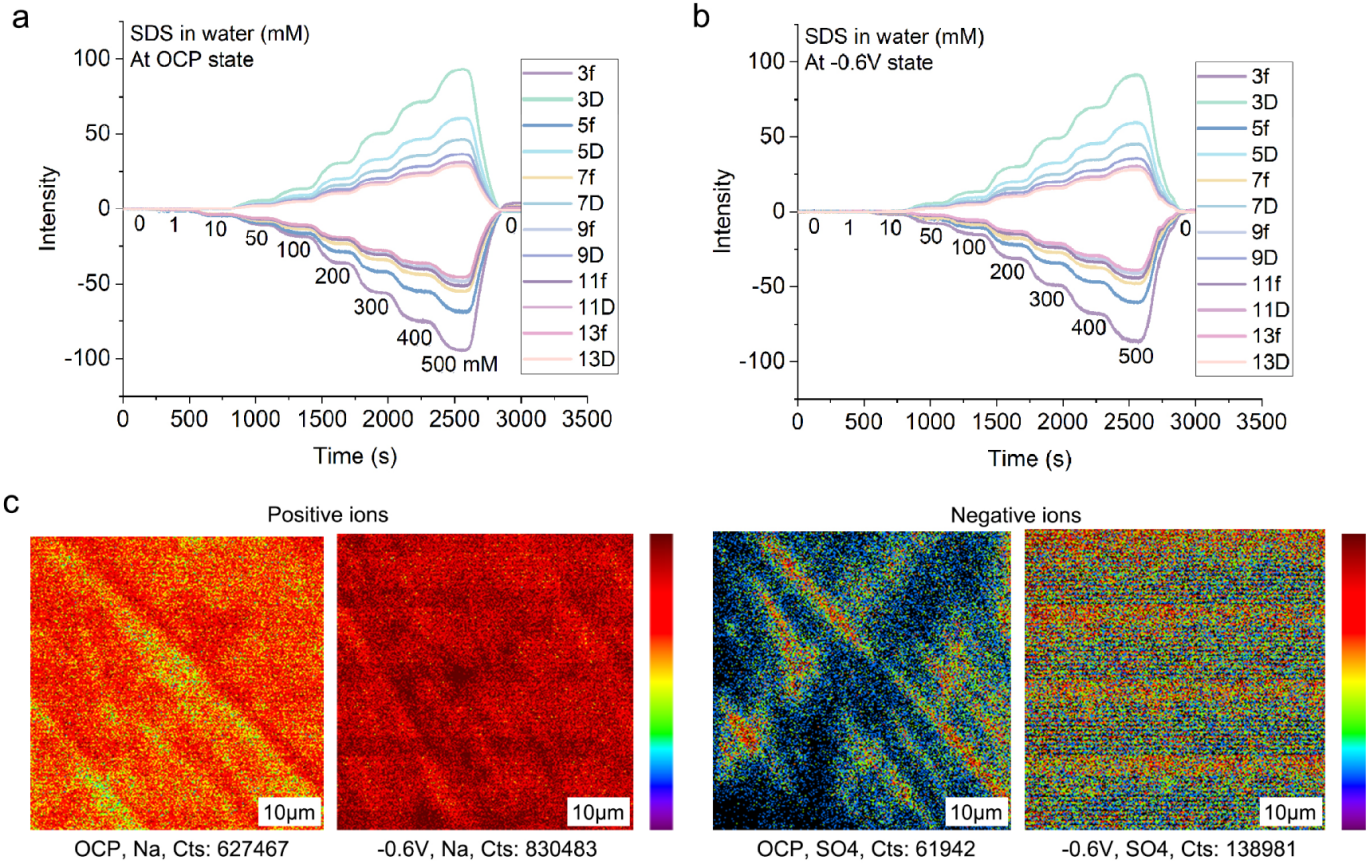
Adsorption film characterizations. The effect of SDS concentration on the change in frequency and dissipation of a gold-coated QCM sensor surface **a.** at the OCP and **b.** at −0.6 V. **c.** ToF-SIMS images of the 52100 steel disc surface after immersing in 500 mM SDS solution with and without applied potentials.

Surfaces of 52100 steel discs which have been submerged in 500 mM SDS solution for one min with and without a negative potential were examined with Time-of-Flight Secondary Ion Mass Spectrometry (ToF-SIMS); see Figure 8c. There is a stronger and more uniform presence of sodium and sulfate ions on the disc that experienced a negative potential. Note that the amount of sodium ions in the total fragments increases from 30% in OCP to 50% when a negative potential is applied, similarly observed in ref 40. This demonstrates that the application of a negative potential on the metal surface can alter the chemical composition and hence the structure of the SDS adsorbed film. This may impact the COF of the steel–steel contacts.

### 3.7 Hypothesis on Impact of Excess Na^+^ in Structure of SDS Adsorbed Film

The adsorption of SDS on metal surfaces is governed by both electrostatic and hydrophobic interactions between the surfactant aggregates, the solvent, and the metal surfaces. The structure of the SDS aggregate depends on its concentration. At a very low concentration, SDS adsorbs as individual molecules. Above 8 mM, SDS hemicylindrical micelles are formed on metal surfaces such as gold and stainless steel.^47^ As all of our solutions are more concentrated than 8 mM, hemicylindrical SDS micellar layers are expected to form in all cases. Note that Na^+^ ions are in the proximity, balancing the surface charges. Based on friction results under the OCP (see Figure 3a), this SDS layer is robust against rubbing and results in low friction in our test conditions when the SDS concentration is above 0.1 mM.

When a negative potential is applied, more sodium ions move closer to the metal surfaces. Yet, the SDS anions, as shown by the QCM results in Figure 8, remain on the surface as an adsorbed layer due to strong hydrophobic interactions. An increase in Na^+^ in the proximity of the surface may, however, affect the structure and morphology of the SDS adsorbed layer, leading to a change in friction. The strong hydrophobic interactions within the SDS layers mean a large excess in Na^+^ is necessary to induce sufficient change in the SDS layers to affect friction. So, we hypothesize that under a negative potential:

- It is the amount of Na^+^, rather than the removal of SDS anions, that governs the morphology of adsorbed SDS layer. More specifically, at a fixed SDS concentration, a critical amount of excess Na^+^ is necessary to induce a sufficient change in the morphology of the SDS adsorbed layer, resulting in a change in friction

Since the amount of Na^+^ near the surface is related to the surface potential, the existence of a critical applied potential for noticeable change in friction in an experiment, as shown in Figure 3f, can be understood as the existence of a critical amount of excess Na^+^. Hence, one would expect the critical value of applied voltage to increase as the concentration of SDS, and hence that of Na^+^ in the bulk solution, decreases. This is indeed the case. Compared to a critical value of −0.4 V for the 500 mM SDS solution (Figure 3f), −0.9 V is needed for the 10 mM solution (see Figure 9b). Note that for 1 mM SDS, even −0.9 V is insufficient to induce a change in friction (Figure 9a). Higher applied voltage was not tested as they are above the electrochemical stable window of this test system. This hypothesis can explain why adding NaCl into more dilute SDS solutions reduces the critical applied voltage: adding NaCl provides the excess Na^+^ needed to generate compositional and morphological changes of the SDS adsorbed layer under the applied voltage. To assess our stated hypothesis, computational studies were conducted, and results are shown in Section 3.8.

**Figure 9:**
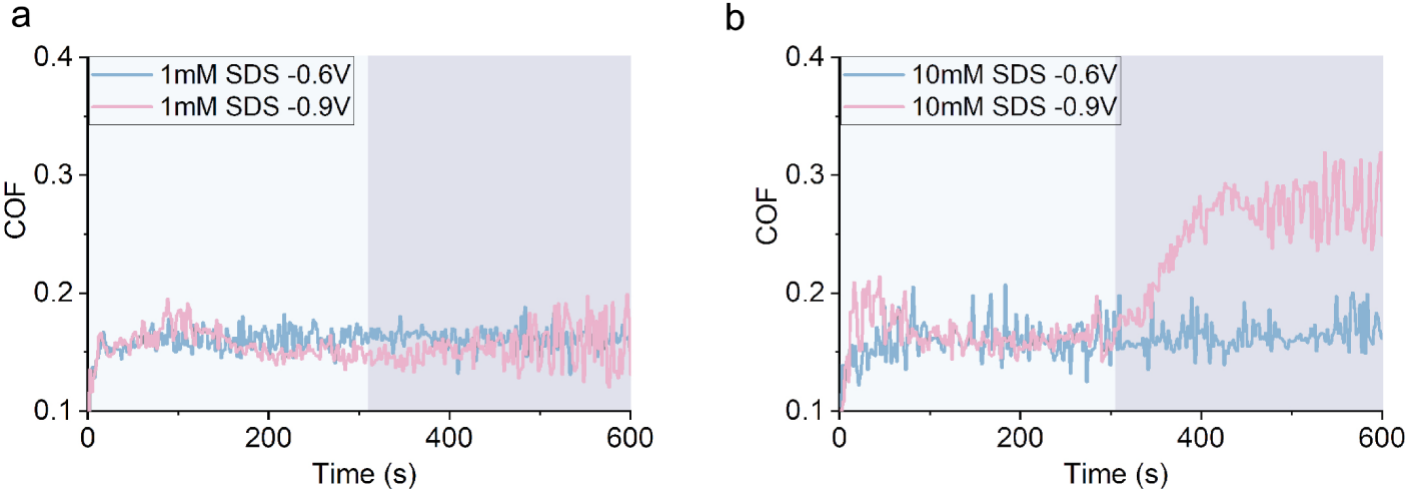
COF of **a.** 1 mM SDS at −0.6 V and −0.9 V. **b.** 10 mM SDS at −0.6 V and −0.9 V.

### 3.8 Effects of Surface Potential on Structure of SDS Adsorbed Layer

MD simulations were conducted to investigate the effect of the surface potential on the structure of SDS adsorbed layers. Simulations of a 50 mM NaCl aqueous solution were first carried out to examine the effect of NaCl alone. With no applied surface potential, the distributions of Na^+^ and Cl^–^ in the proximity of the surface are similar on both surfaces. When a potential difference of 2 V is applied, the top and bottom surfaces have a surface potential of −1 V and +1 V, respectively. A large amount of Na^+^ is attracted to the negatively charged surface, forming layers (Figure S9). The IHP now contains a significantly higher concentration of Na^+^ than the bulk concentration. The concentration of Na^+^ of subsequent layers decreases away from the surface until the bulk concentration is reached. At the same time, the amount of Cl^–^ in the IHP region on the negatively charged surface is lower than that of the bulk concentration. The reverse is observed on the positively charged surface, where Cl^–^ ions form layers in its proximity, while Na^+^ ions are depleted. This induced fluid layer structure in the proximity of a solid surface is commonly seen,^68^ and these results match observations from the literature.^57^ The surface charge-induced fluid layer structure has been speculated to impact the friction of charged rubbing surfaces.^69^ In this work, however, the layering of ions on surfaces under an applied potential in NaCl_(aq)_ has a limited effect on friction (see Figure 7a and b). This is probably because the roughness of our surface is comparable to the thickness of the double layers. Together with the high applied pressure, the impact of these layers on friction is limited.

Simulations of SDS solutions show that layered structures are formed near the surfaces with or without applied surface potentials; see peaks of the population density functions (PDFs) in Figure 10. Under zero applied potential, SDS anions form hemicylindrical micelles on surfaces; the hydrophobic tails form the core of the micelles and are shielded from water by the negatively charged headgroup (Figure 10a). Na^+^ ions are in the proximity of the surface to achieve charge balance. As a result, the concentrations of both SDS and Na^+^ ions on the surface are high. Note that the PDFs of the SDS tail group, its negatively charged headgroup, and Na^+^ follow each other closely.

**Figure 10:**
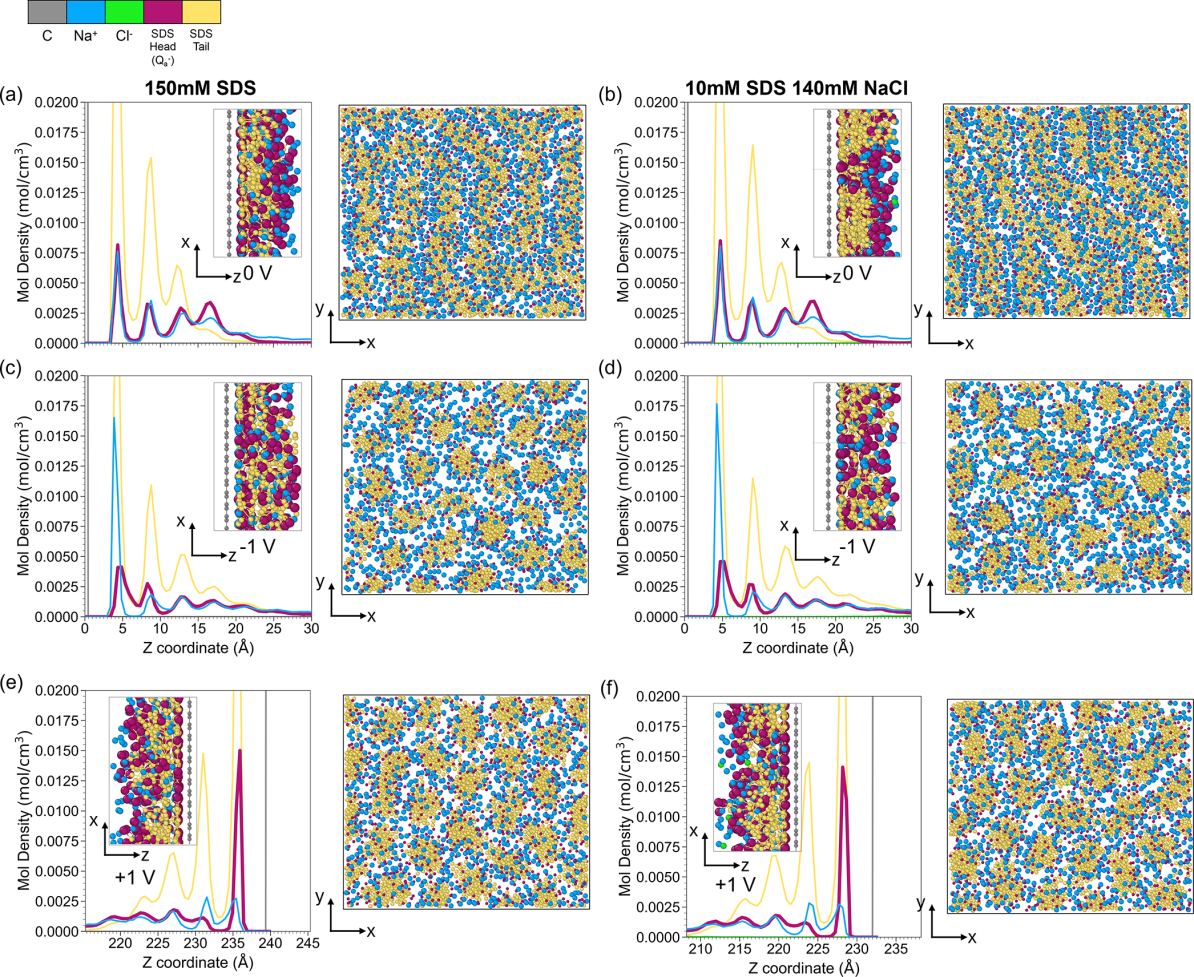
Simulation results of SDS solutions on surfaces with or without applied potentials. Population density functions of SDS, NaCl, and structures of adsorbed SDS layers of **a.** 150 mM SDS at the OCP, **b.** 10 mM SDS + 140 mM NaCl at the OCP, **c.** 150 mM SDS at −1 V, **d.** 10 mM SDS + 140 mM NaCl at −1 V, **e.** 150 mM SDS at +1 V, and **f.** 10 mM SDS + 140 mM NaCl at +1 V.

When the surface potential is −1 V (Figure 10c; see also Figure S10a and S11), the peak of the PDF of Na^+^ next to the surface increases substantially in magnitude and has shifted closer to the surface, supporting the results from ToF-SIMS (Figure 8c). The amount of SDS tail groups is reduced only slightly because their adsorption is governed mainly by hydrophobic interactions between the hydrocarbon chains, with their anionic headgroups shifted slightly outward. This leads to a significant change in the ratio of the surface concentration of Na^+^ and the anionic SDS molecules (see Figure S10a). The structure of the adsorbed SDS layer changes from hemicylindrical at 0 V to hemispherical at −1 V; see the *x*–*y* view of the adsorbed layers in Figure 10a and c. The higher surface concentration of Na^+^ screens the electrostatic repulsion between the anionic SDS headgroups, enabling more of them to come closer together and form hemispheres rather than hemicylinders. The size distribution of these aggregates has also changed substantially (Figure S12), with the hemispherical micelles being smaller and more uniform in size. These structural changes correspond to the increased friction at −1 V compared to 0 V. It is not obvious why hemispherical surface micelles show higher friction than hemicylinders in a macroscale rubbing contact. One possibility is that hemicylinders provide more even load distribution and thus lower peak pressures at asperities than hemispheres, in analogy to the difference between macroscale line and point contacts.

The effect of a positive surface potential on the PDFs of Na^+^ and SDS anions is the reverse of what is observed with a negative surface potential; see Figure 10e. The peak of the PDF of Na^+^ next to the surface decreases slightly in magnitude (see also Figure S10c) and has shifted away from the surface. However, the amount of SDS tail groups remains unaffected (see Figure S10c) probably because its surface coverage is already high even at the OCP. Overall, the ratio between the number density of anions and Na^+^ is relatively constant with the applied positive potential. Thus, the structure of the adsorbed SDS layer undergoes less change in this case (Figure 10e; also compare the aggregate size distribution and structures under +1 V in Figure S13a and b and −1 V in Figure S12a and b). This may explain why the COF is insensitive to the applied positive potential.

Friction results in Figure 3b and Figure 5b show that adding NaCl (>100 mM) increases the sensitivity of friction of 10 mM SDS solution to applied negative potential. In fact, the friction of 150 mM SDS solution and 10 mM SDS + 140 mM NaCl respond to a negative surface potential similarly. Simulations show that 10 mM SDS + 140 mM NaCl solution (resulting in a total sodium ion concentration of 150 mM) has similar PDFs and adsorbed micellar structures to those of 150 mM SDS solution; see Figure 10a-f (see also Figure S10, S12 and S13). This shows that the structural change of the adsorbed SDS micelles induced by excess Na^+^ under a negative surface potential leads to a change in friction. This structural change, however, requires a large amount of Na^+^ to be attracted near the surface, probably due to the strong hydrophobic interactions between SDS and water. Without sufficient Na^+^, the applied surface potential cannot induce a structural change of the SDS adsorbed layer.

Previous reports have shown that applied potentials can influence the rates of adsorption and desorption of cations and anions.^38^ The stability of the SDS aggregate structure on a negatively charged surface is investigated by observing its temporal evolution after the surface potential is turned off (Figure 11). At −1 V, adsorbed SDS exhibits a hemispherical micellar structure. Intuitively, when the surface potential is returned to zero, Na^+^ may migrate away from the surface, and hence, the adsorbed aggregates may revert back to hemicylindrical micelles. This transition, however, is not instantaneous (Figure 11b). When the potential is switched from −1 V to +0.5 V; the hemispherical micellar structure reverts back to hemicylindrical quickly (Figure 11c), as the Na^+^ ions are being repelled by the positive surface potential. This behavior closely mirrors the effect of switching potential on the COF (Figure 7d,e). The results confirm the role of Na^+^ ions in the determination of the morphology of the absorbed layer, which subsequently impacts the friction of rubbing contacts in SDS aqueous solutions.

**Figure 11:**
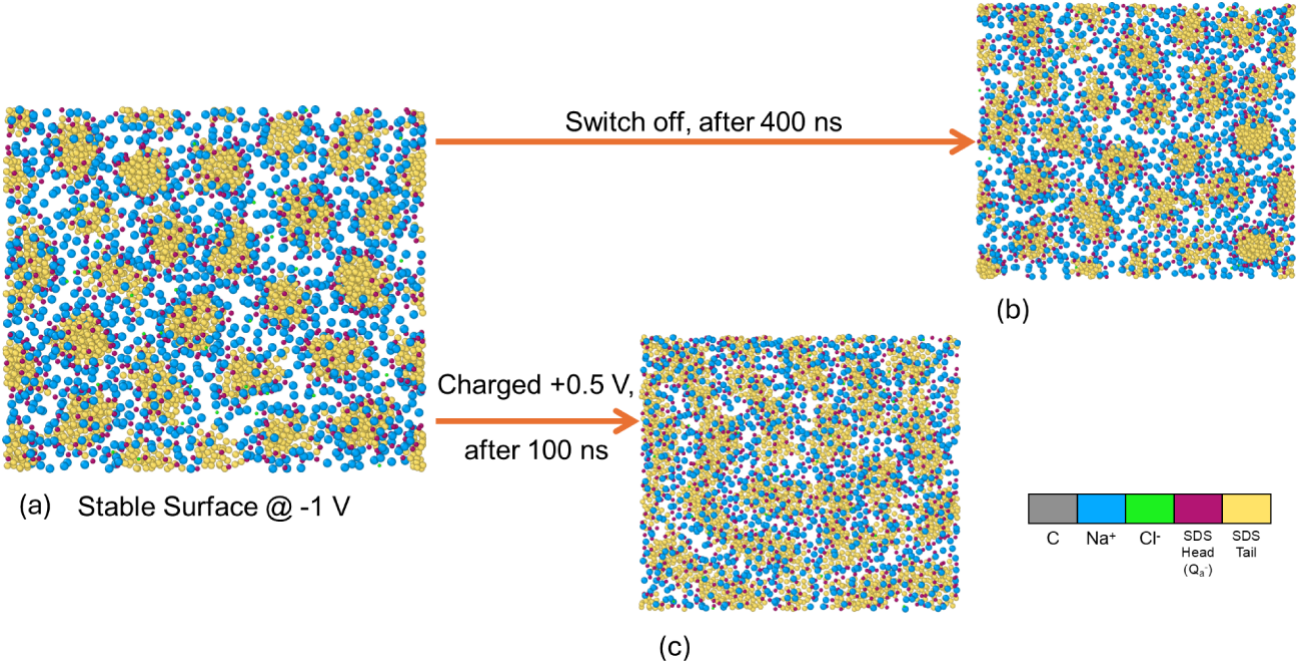
Morphology evolution of the adsorbed SDS layer: **a.** at −1 V; **b.** 400 ns after the negative −1 V surface potential in **a.** is switched off; **c.** the surface potential is switched from −1 V in **a.** to 0.5 V and applied for 100 ns.

## 4 Conclusions

The effect of applied potential on the lubrication performance of SDS aqueous solutions for steel–steel contact is investigated through experiments and computational modeling. SDS forms an adsorbed layer on steel surfaces, resulting in a low friction. The structure of the adsorbed SDS aggregates is governed by electrostatic and hydrophobic interactions between the Na^+^ ions, SDS anions, and water molecules. At open circuit potential, hemicylindrical SDS micelles are formed on the rubbing surfaces. The effect of changing the surface potential is mainly to change the distribution of Na^+^ on the surface, while the distribution of SDS anions remains relatively unaffected due to the strong hydrophobic interactions between the SDS tail groups. A significant change in SDS micellar structure (from hemicylindrical to hemispherical) and an increase in the friction coefficient are then observed when:

- An abundance of Na^+^ is available: this occurs with concentrated SDS solutions or relatively dilute SDS solutions with the addition of NaCl. The critical Na^+^ concentration is ∼100 mM.
- A high negative surface potential is applied: this, together with a high concentration of Na^+^, allows adequate amount of Na^+^ to accumulate on surface.

Note that only small amounts of SDS are needed to achieve high SDS surface coverage due to their strong hydrophobic interactions. Thus, applying a positive surface potential cannot attract more SDS and has little effect on changing the structure of the adsorbed SDS aggregate. This explains the insensitivity of friction in SDS solutions under a positive surface potential.

In summary, the additive and salt concentrations in the lubricant are crucial in determining the triboelectrical response of SDS aqueous solutions. Under a negative applied potential, there are two coupled effects: (i) enrichment of cations at the interface and (ii) restructuring of the adsorption layer. Specifically, the availability of sodium ions needs to reach a certain threshold to screen repulsive electrostatic interactions between the negatively charged SDS headgroups and promote a transition from hemicylindrical surface micelles to hemispherical ones. The latter structure shows a higher macroscale friction. The structural transition can be promoted either by elevating the concentration of SDS or through the incorporation of additional sodium salts in the aqueous lubricants.

This study highlights the importance of hydrophobic interactions in achieving electrotunable friction and is likely to be relevant to surfactants and ionic liquids, where hydrophobic interactions are likely to play a role. In this case, the role of small, more electro-responsive cations may hold the key in turning the tribological response of the additives. To design an electro-responsive surfactant-type additive, its hydrophobic interactions should be overcome relatively easily with an applied potential. This, however, should not be detrimental to the formation of protective layers. We hope that an understanding of this mechanism may contribute to the development of advanced electro-responsive additives, thereby catalyzing progress in the field of active tribology.

## Supplementary Material



## Data Availability

The data that support the findings of this study are available from the corresponding author upon reasonable request.

## Notes

Detailed information about the modeling approach and computational methods, a schematic diagram of the two-electrode systems with HFRR, the wear track corresponding to Figure 3d, wear measurements under OCP and −0.6 V, friction of SDS solutions using a two-electrode system, COF of 500 mM SDS solution using a stainless steel disc under −0.6 V, COF performances of the steel/ZrO_2_ rubbing pair using SDS as an aqueous lubricant additive under −0.6 V, properties of SBS solutions, adsorption of different SDS concentrations on the iron oxide surface at the OCP state, the behaviors of 50 mM NaCl on the rubbing surface, surface coverage of SDS solution, population density functions of SDS species in 150 mM SDS solutions, and the size distribution of aggregates (PDF)
