# Supplementary material for: Electrically Tunable Friction through Surface Adsorption Layer Restructuring
Source: ACS Appl Mater Interfaces. 2025 Dec 17;17(52):70994–1007. doi: 10.1021/acsami.5c20376 (PMC12766684; doi:10.1021/acsami.5c20376)
Supplement: Supplementary file 1 [file am5c20376_si_001.pdf]

Supporting information

Electrically Tunable Friction through Surface Adsorption Layer Restructuring

Yun Zhao[a,c], Zhaoran Zhu[a,c], Jie Zhang[a], Erik Weiand[a], Chao Wang[b], James P. Ewen[a], Daniele Dini[a], Hugh A. Spikes[a], & Janet S.S. Wong[a, *]

[a] Department of Mechanical Engineering, Imperial College London, London SW7 2AZ, UK

[b] State Key Laboratory of Solid Lubrication, Lanzhou Institute of Chemical Physics, Chinese Academy of Sciences, Lanzhou 730000, China

[c] These authors contributed equally to this study.

*Corresponding author: j.wong@imperial.ac.uk

Simulation details

Most of the MARTINI force field parameters used in this study has been reported in our previous studies.[1,2] The non-bonded interaction between polarizable water bead and the graphene C_1 bead is tuned to be around 70% of the original interaction strength to obtain a hydrophobic surface. The non-bonded interaction between SDS tail C_1 bead and the graphene C_1 bead is reduced to 60% of the original interaction strength as reported by Wallace and Sansom,[3] to preserve the stable structure. All the force field parameters are summarized in **Table S1**.

The non-bonded interactions between MARTINI beads are cut off at a distance $r_{c,cut}$ of 12 Å.[4] The shifted Lennard-Jones potentials are used for non-aqueous uncharged beads, and smoothly shifted to zero between the cut-off radius $r_{LJ,cut}$ = 9 Å and $r_{c,cut}$.[4] All the remaining non-bonded interactions are treated as described in the polarizable MARTINI water model.[5] Short-range electrostatic interactions between the charged beads are described by the coulombic potentials with a switching radius of $r_{c,cut}$.[4] Long-range electrostatic interactions are calculated using the particle–particle particle–mesh (PPPM) method[6] for the ELECTRODE package (pppm/electrode)[7] at a relative energy tolerance of 10^{-5}. Bonds are treated using weak harmonic potentials as in the original MARTINI framework.[4] The bond lengths in the water units are constrained to r = 1.4 Å using the SHAKE algorithm.[5,8]

Table S1 Non-bonded interactions

$$E_{LJ} = 4\varepsilon\left[\left(\frac{\sigma}{r}\right)^{12} - \left(\frac{\sigma}{r}\right)^{6}\right] + S_{LJ}(r)$$

**Bead A**	**Bead B**	**Type** [4, 5]	**ε (kcal/mol)**	**s (Å)**
**C_1**	C_1	IV	0.8361	4.7
**C_1**	W	0.7*VIII	0.3298	4.7
**C_1**	Q_d	VII	0.5497	4.7
**C_1**	Q_a	VII	0.5497	4.7
**C_1**	C_1(SDS)	0.6*IV	0.5017	4.7
**W**	W	-	0.9556	4.7
**W**	Q_d	I	1.1945	4.7
**W**	Q_a	I	1.1945	4.7
**W**	C_1(SDS)	0.95*VIII	0.4539	4.7
**Q_d**	Q_d	IV	0.8361	4.7
**Q_d**	Q_a	III	0.9556	4.7
**Q_a**	Q_a	IV	0.8361	4.7
**C_1(SDS)**	C_1(SDS)	IV	0.8361	4.7

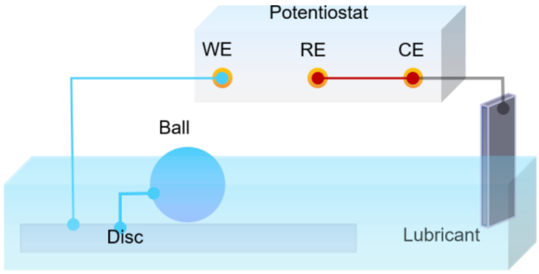

Figure S1. Schematic diagram of the two-electrode systems with HFRR.

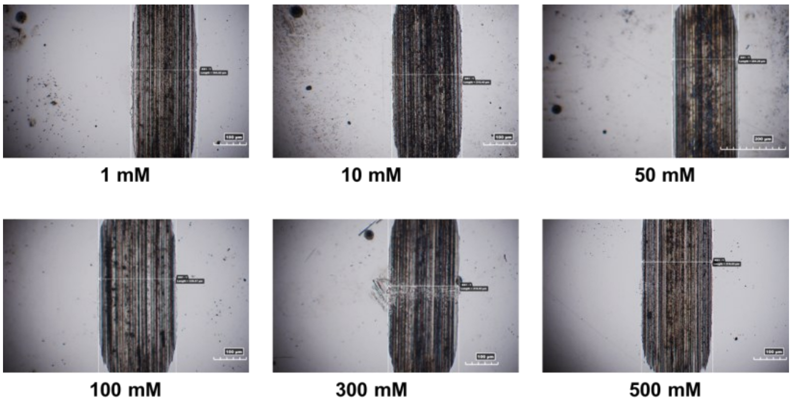

Figure S2. Wear track corresponding to Figure 3d.

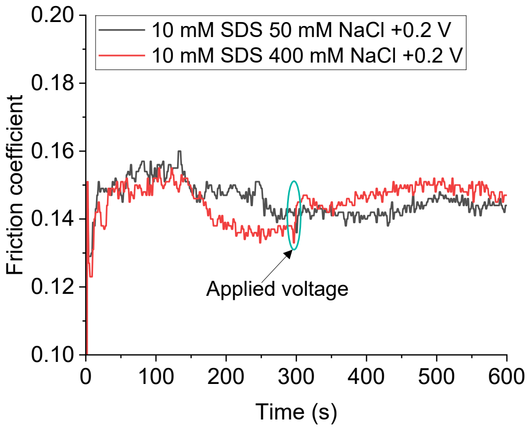

Figure S3 The effect of positive potential on COF of 10 mM + NaCl

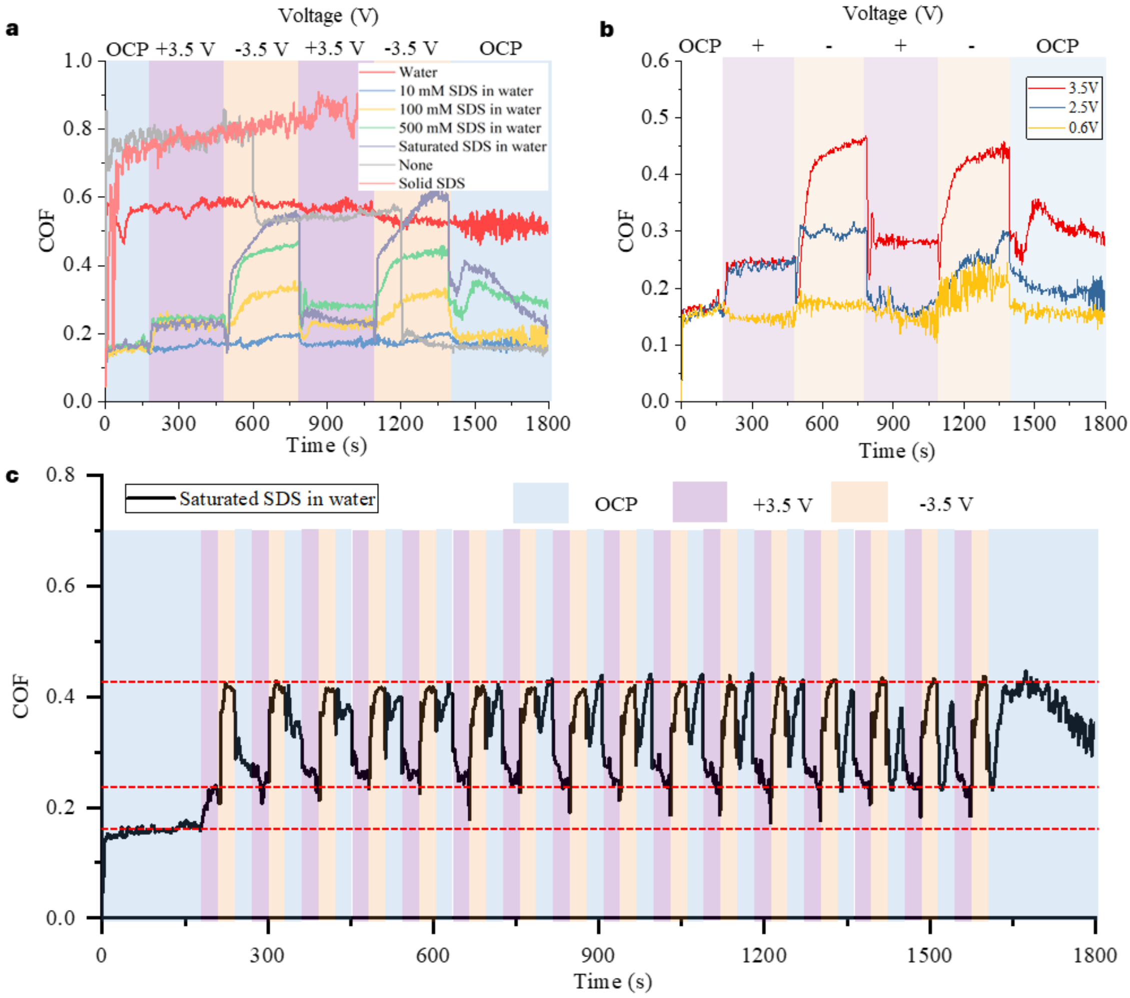

Figure S4. Friction of SDS solutions using a two-electrode system. a. COF of SDS solutions of various concentrations under ±3.5V. b. COF of 500mM SDS solution under various voltage conditions. c. COF of 500mM SDS solution under ±3.5V in cyclic conditions.

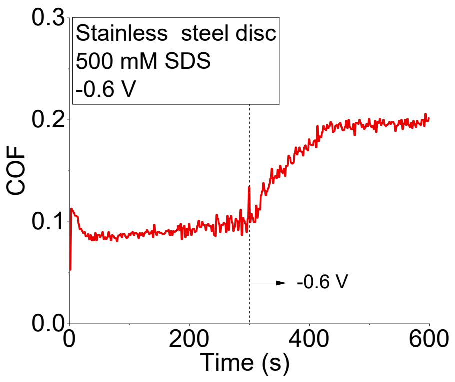

Figure S5. COF in 500 mM SDS solution using steel ball and stainless steel disc under -0.6 V.

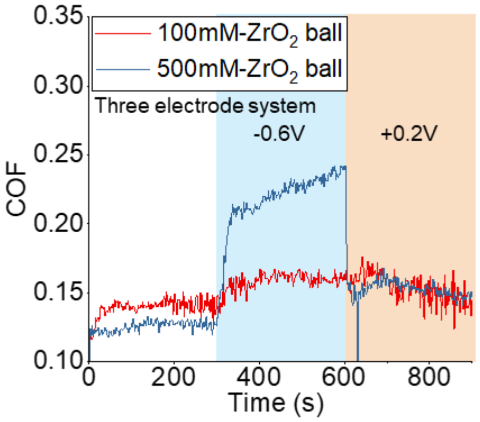

Figure S6. COF performances of steel/ZrO_2 rubbing pair using SDS as aqueous lubricant additive under -0.6V.

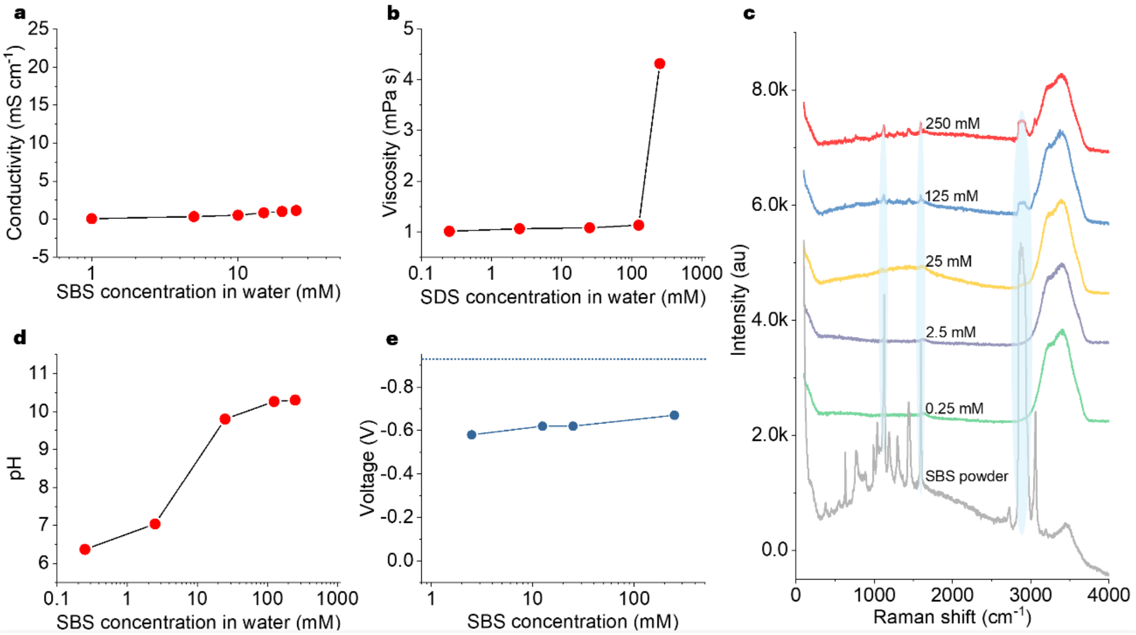

Figure S7. Properties of SBS solutions. a. Conductivity. b. Viscosity. c. Raman spectrum. d. pH. e. Open circuit potential (The dotted line represents the set testing potential).

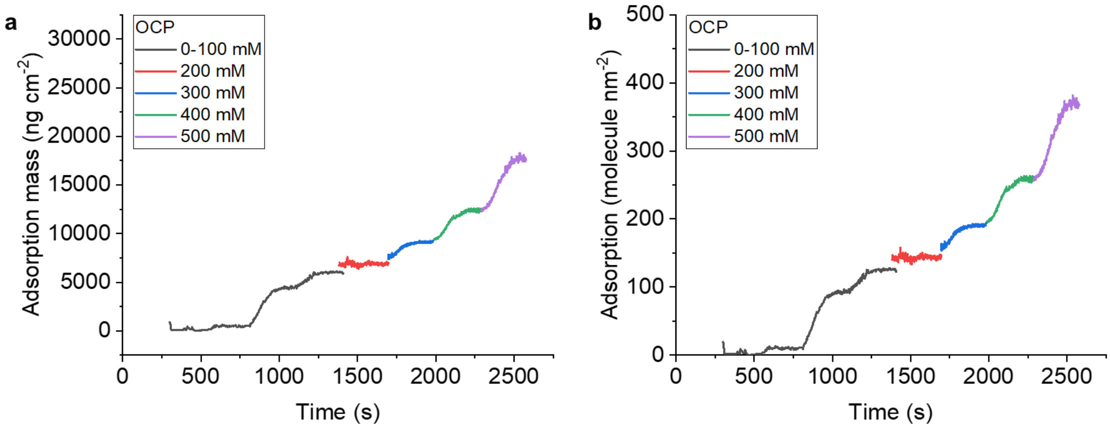

Figure S8. Adsorption of different SDS concentration on iron oxide surface at OCP state. a. Adsorption mass. b. Adsorption molecule.

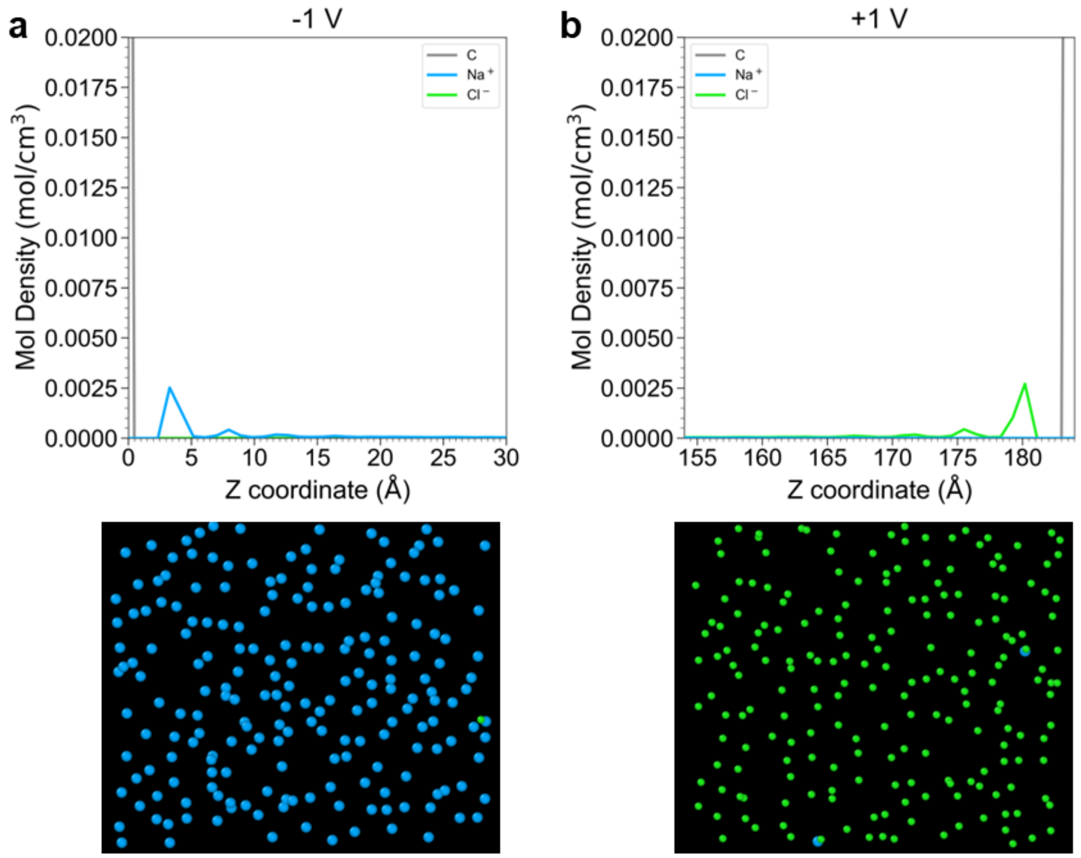

Figure S9. The behaviours of 50 mM NaCl on rubbing surface. Top and bottom surfaces correspond to z = 200 and 0 Å respectively. a. Negative surface showing the accumulation of sodium ions. b Positive surface showing the accumulation of chloride ions.

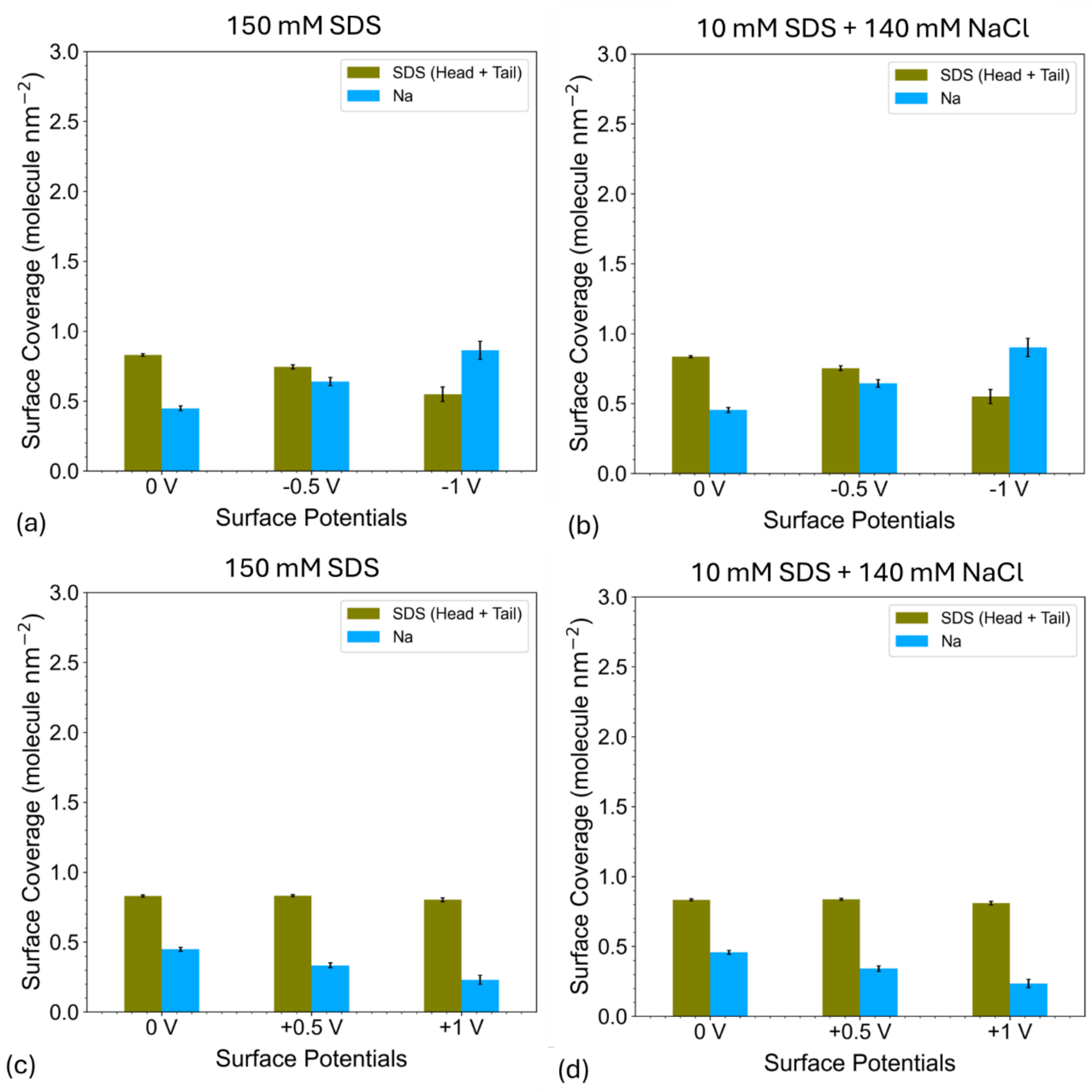

Figure S10. Surface coverage of (a) 150 mM SDS solution under negative voltage, (b) 10 mM SDS + 140 mM NaCl solution under negative voltage, (c) 150 mM SDS solution under positive voltage, and (d) 10 mM SDS + 140 mM NaCl solution under positive voltage. Only the first 6 Å of liquid from the surface. This corresponds to the inner Helmholtz plane.

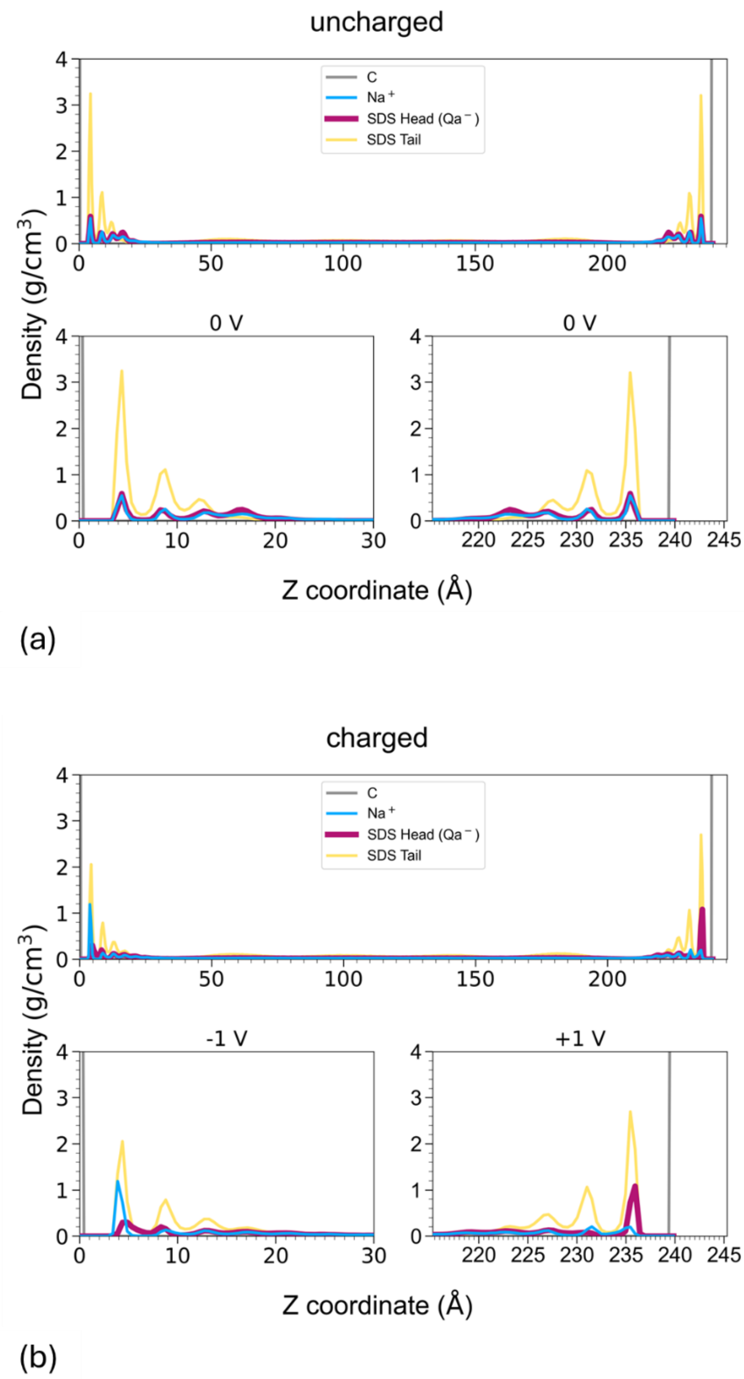

Figure S11. Population density functions of SDS species in 150 mM SDS solutions. (a) without applied potential (b) under the application of 2 V, the surface potential thus is -1 V and +1 V.

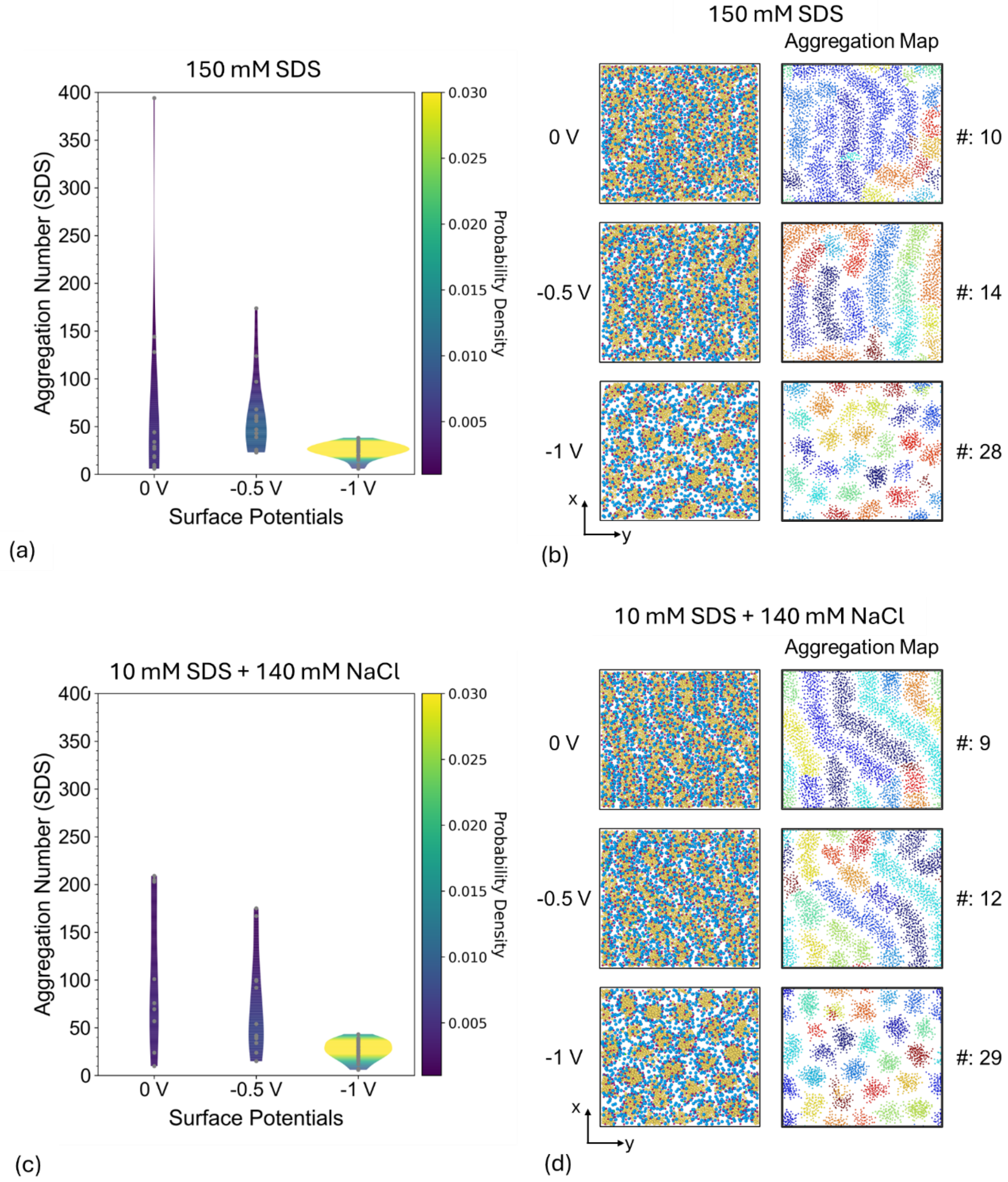

Figure S12. The size distribution of aggregates and the x-y views of the adsorbed SDS layers formed under negative surface potential in (a) and (b) 150 mM SDS; (c) and (d) 10 mM SDS+140 mM NaCl. Aggregation maps highlight individual micelles.

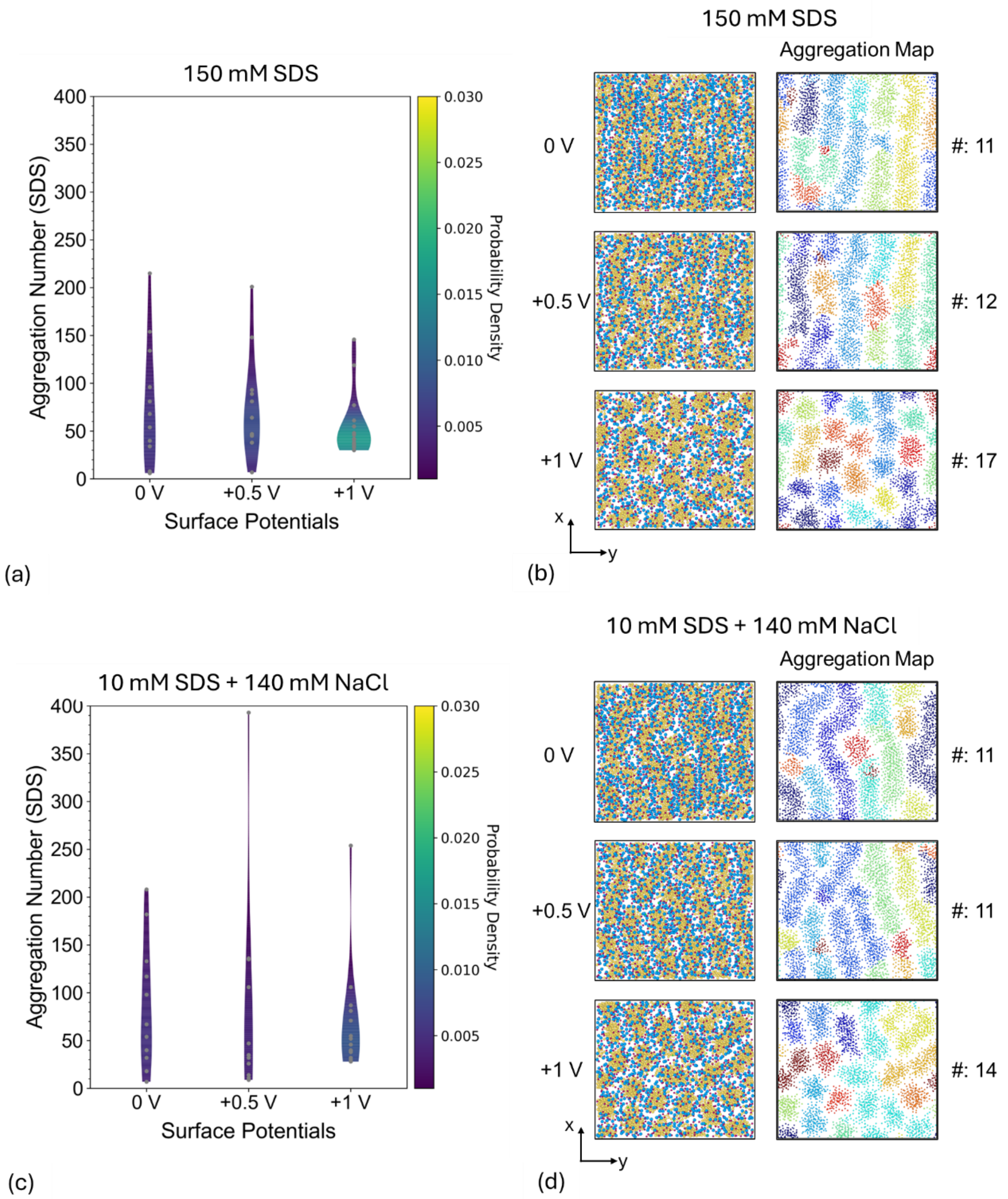

Figure S13. The size distribution of aggregates and the x-y views of the adsorbed SDS layers formed under positive surface potentials in (a) and (b) 150 mM SDS; (c) and (d) 10 mM SDS+140 mM NaCl. Aggregation maps highlight individual micelles.

References

(1) Weiand, E.; Rodriguez-Ropero, F.; Roiter, Y.; Koenig, P. H.; Angioletti-Uberti, S.; Dini, D.; Ewen, J. P. Effects of Surfactant Adsorption on the Wettability and Friction of Biomimetic Surfaces. *Physical Chemistry Chemical Physics* 2023, *25* (33), 21916–21934. https://doi.org/10.1039/D3CP02546B.

(2) Weiand, E.; Ewen, J. P.; Koenig, P. H.; Roiter, Y.; Page, S. H.; Angioletti-Uberti, S.; Dini, D. Coarse-Grained Molecular Models of the Surface of Hair. *Soft Matter* 2022, *18* (9), 1779–1792. https://doi.org/10.1039/D1SM01720A.

(3) Wallace, E. J.; Sansom, M. S. P. Carbon Nanotube/Detergent Interactions via Coarse-Grained Molecular Dynamics. *Nano Lett* 2007, *7* (7), 1923–1928. https://doi.org/10.1021/NL070602H/SUPPL_FILE/NL070602HSI20070314_051723.PDF.

(4) Marrink, S. J.; Risselada, H. J.; Yefimov, S.; Tieleman, D. P.; De Vries, A. H. The MARTINI Force Field: Coarse Grained Model for Biomolecular Simulations. *Journal of Physical Chemistry B* 2007, *111* (27), 7812–7824. https://doi.org/10.1021/JP071097F/ASSET/IMAGES/LARGE/JP071097FF00007.JPEG.

(5) Yesylevskyy, S. O.; Schäfer, L. V.; Sengupta, D.; Marrink, S. J. Polarizable Water Model for the Coarse-Grained MARTINI Force Field. *PLoS Comput Biol* 2010, *6* (6), e1000810. https://doi.org/10.1371/JOURNAL.PCBI.1000810.

(6) Hockney, R. W.; Eastwood, J. W. *Computer Simulation Using Particles*; CRC Press, 1989. https://doi.org/10.1201/9780367806934.

(7) Ahrens-Iwers, L. J. V.; Meißner, R. H. Constant Potential Simulations on a Mesh. *J Chem Phys* 2021, *155* (10). https://doi.org/10.1063/5.0063381.

(8) Ryckaert, J. P.; Ciccotti, G.; Berendsen, H. J. C. Numerical Integration of the Cartesian Equations of Motion of a System with Constraints: Molecular Dynamics of n-Alkanes. *J Comput Phys* 1977, *23* (3), 327–341. https://doi.org/10.1016/0021-9991(77)90098-5.
